# HDAC2 as a therapeutic target in bladder cancer: insights into the altered epigenetic regulation and lysine lactylation

**DOI:** 10.1186/s13046-025-03610-5

**Published:** 2025-12-17

**Authors:** Guanghui Xu, Shuo Liang, Ganlin Hu, Wei Zhao, Yuqin Li, Minghao Zheng, Zhigang Wu, Tianlei Xie, Shuting Fang, Shan Peng, Yongming Deng, Yihua Zhou, Hongqian Guo, Junlong Zhuang, Wenli Diao

**Affiliations:** 1 Department of Urology, Nanjing Drum Tower Hospital, Affiliated Hospital of Medical School, Nanjing University, Nanjing, China; 2 Institute of Urology, Nanjing University, Nanjing, China; 3 Department of Urology, Nanjing Drum Tower Hospital Clinical College of Nanjing Medical University, Nanjing, China; 4 Department of Urology, Nanjing Drum Tower Hospital Clinical College of Nanjing University of Chinese Medicine, Nanjing, China; 5 Department of Clinical Biochemistry, School of Laboratory Medicine, Chengdu Medical College, Chengdu, China; 6 Clinical Laboratory, Clinical Medical College, The First Affiliated Hospital of Chengdu Medical College, Chengdu, China; 7 Department of Urology, Affiliated Hospital of Nanjing University of Chinese Medicine, Jiangsu Province Hospital of Chinese Medicine, Nanjing, China; 8 Department of Pathology, Nanjing Drum Tower Hospital, Affiliated Hospital of Medical School, Nanjing University, Nanjing, China; 9 Department of Infectious Diseases, Nanjing Drum Tower Hospital, Affiliated Hospital of Medical School, Nanjing University, Nanjing, China

**Keywords:** Bladder cancer, Epigenetic regulation, HDAC2, Post-translational modifications, Lactylation

## Abstract

**Background:**

The pathogenesis of bladder cancer (BCa) is driven in part by aberrant epigenetic regulation, most notably the dysregulated expression of histone deacetylases (HDACs). As a class I HDAC, HDAC2 is often overexpressed in cancers and promotes malignancy through diverse mechanisms. Given its broad oncogenic role, an in-depth investigation of its specific functions in epigenetic and post-translational regulation within BCa holds significant promise for developing novel precision therapies.

**Methods:**

*In vitro* functional assays, including CCK-8, colony formation, transwell and apoptotic assays, as well as *in vivo* assays in a nude mouse subcutaneous tumor model, were performed to assess the oncogenic and drug-resistant effects of HDAC2. RNA-seq and ATAC-seq were employed to analyze the epigenetic regulatory mechanisms of HDAC2. Combined proteome, lactylome and acetylome analysis of control and HDAC2-overexpressing BCa cells were conducted to map the global profiling of protein lysine acetylation (Kac) and lactylation (Kla).

**Results:**

*In vitro* and *in vivo* experiments confirmed that HDAC2 overexpression significantly promoted proliferation, metastasis and chemoresistance of BCa. Integrated RNA-seq and ATAC-seq analysis revealed that HDAC2 overexpression led to significant epigenetic alternations, and knockdown of its downstream GRIK2 significantly reversed the oncogenic effects of HDAC2. We screened class I HDACs for their impact on Kac and Kla in BCa cells and found that HDAC2 most significantly reduced global Kla levels. Subsequent proteomic analysis of HDAC2-overexpressing cells identified 528 differentially regulated Kla proteins (encompassing 683 sites) and 1,129 differentially regulated Kac proteins (encompassing 1,458 sites). Notably, DHX15 in the splicesome pathway emerged as the most prominent HDAC2-regulated lactylated protein in the absence of concurrent Kac alterations. Moreover, HDAC2 promoted BCa malignancy through the downregulation of DHX15 Kla and the subsequent modulation of RPL9 splicing.

**Conclusion:**

Collectively, these findings suggest the pivotal role of HDAC2 in epigenetic modulation and lysine lactylation, and underscore HDAC2 as a promising therapeutic target in BCa.

**Supplementary Information:**

The online version contains supplementary material available at 10.1186/s13046-025-03610-5.

## Background

Bladder cancer (BCa) remains a major global health challenge, ranking as the ninth most common malignancy worldwide, with 573,000 new cases and 213,000 deaths annually [1]. It exhibits a strong male predominance (3–4 times higher incidence in men than women), with peak occurrence after age 60–70. Approximately 75% of cases are non-muscle-invasive (NMIBC), typically managed with transurethral resection and intravesical therapy (e.g., BCG or chemotherapy), while muscle-invasive (MIBC) and metastatic disease require radical cystectomy, radiotherapy, or systemic therapies [2]. Despite treatment advances, the 5-year survival remains highly stage-dependent, ranging from >80% for localized disease to < 40% for metastatic cases [3]. Recent therapeutic innovations, such as immune checkpoint inhibitors (e.g., pembrolizumab) and antibody-drug conjugates (e.g., enfortumab vedotin), have improved outcomes in advanced disease [4]. However, high intertumoral heterogeneity leads to vastly divergent responses among patients, which underscores the urgent need to explore novel pathogenic mechanisms and to develop strategies for improving sensitivity to standard therapies.

BCa exhibits extensive epigenetic alterations, including DNA methylation, histone modifications, and non-coding RNA dysregulation [5]. Notably, mutations in chromatin remodelers (*KDM6A*, *KMT2D*, *ARID1A*, *EP300*) occur in ~ 80% of cases [6], driving transcriptional dysregulation and tumor progression. Emerging evidence highlights novel protein post-translational modifications (PTMs), such as lactylation (Kla) [7], S-palmitoylation [8] and SUMOylation [9] in BCa pathogenesis. These aberrant PTMs offer therapeutic targets, including HDAC inhibitors and EZH2 modulators, with ongoing clinical trials exploring combination strategies [10, 11].

The histone deacetylase (HDAC) family plays a pivotal role in epigenetic regulation by modulating chromatin structure and gene expression. Dysregulation of HDAC activity is implicated in various cancers, including BCa, where aberrant HDAC expression contributes to tumor progression, immune evasion, and therapeutic resistance [12]. In BCa, HDACs influence key oncogenic pathways, such as cell cycle progression, apoptosis evasion, and epithelial-mesenchymal transition (EMT), making them attractive therapeutic targets [10, 13]. Among HDACs, HDAC2 emerges as a critical player due to its multifaceted roles in cancer biology. HDAC2 regulates cell proliferation, apoptosis, and DNA damage response by modulating key substrates such as p53 and MYC [14]. In colorectal cancer, inhibition of HDAC2 promotes the NLRP3/GSDMD-mediated pyroptosis, thereby enhancing chemosensitivity [15]. Consequently, the involvement of HDAC2 in epigenetic silencing of tumor suppressors and its overexpression in aggressive cancers highlight its potential as a therapeutic target.

In recent years, Kla, a novel PTM derived from lactate, has emerged as a critical regulatory mechanism linking cellular metabolism to epigenetic and functional reprogramming in diseases, particularly cancer [16]. Initially identified on histones (e.g., H3K18la, H4K12la), Kla has since been found to extensively modify non-histone proteins, influencing diverse biological processes such as DNA repair [17, 18], metabolic adaptation [19], immune evasion [20], and stemness maintenance [21]. The lactylome, which presents the comprehensive profiling of Kla sites, has been propelled by advances in high-resolution mass spectrometry. Studies have revealed that Kla is dynamically regulated by “writers” (e.g., p300, KAT8, TIP60) [18, 22, 23], “erasers” (e.g., HDAC1–3, SIRTs) [24, 25], and “readers” (e.g., Brg1) [26], forming a tripartite system that integrates metabolic and epigenetic signaling. For instance, in hepatocellular carcinoma, global lactylome analysis uncovered 9,275 sites, with AK2-K28la suppressing enzymatic activity to drive tumor proliferation [19]. Similarly, in colorectal cancer, KAT8-mediated eEF1A2-K408la enhances protein synthesis, promoting carcinogenesis [23]. Nevertheless, the landscape of enzyme-regulated Kla and acetylation (Kac) remains largely uncharacterized, nor has it been reported in BCa.

In this study, we aimed to investigate the function of HDAC2 in BCa and its underlying regulatory mechanisms. First, we examined the clinical relevance of HDAC2 in BCa utilizing patient samples and public datasets. Subsequently, through *in vivo* and *in vitro* functional experiments, we demonstrated that HDAC2 overexpression significantly promoted malignant behaviors such as proliferation, migration, and chemotherapy resistance in BCa. Additionally, integrated RNA-seq and ATAC-seq analyses revealed that HDAC2 exerted its epigenetic regulatory function by altering chromatin accessibility to enhance the transcription of the downstream gene *GRIK2*. Finally, we mapped the global regulatory landscape of HDAC2 in modulating Kla and Kac modifications in BCa, demonstrating that HDAC2-mediated delactylation significantly altered the function of key proteins in the splicesome pathway. These findings suggest that HDAC2 is a promising therapeutic target for intervention in BCa.

## Methods

### Cell lines and culture conditions

UM-UC-3 and T24 cell lines were obtained from ATCC. UM-UC-3 cells were grown in MEM medium containing 10% fetal bovine serum (FBS), while T24 cells were maintained in RPMI 1640 medium supplemented with 10% FBS. All cell lines were incubated at 37 °C in a humidified atmosphere with 5% CO_2_.

### Patients and clinical samples

Human samples were collected following approval from the Medical Ethics Committee of Nanjing Drum Tower Hospital, with all participants providing written informed consent in compliance with the Declaration of Helsinki.

### Plasmids construction, DNA transfection, virus packaging and lentiviral infection

The cDNA was cloned into Flag-tagged vectors (pcDNA3.1-3×Flag-C) and HA-tagged vectors (pcDNA3.1-3×HA-C). Cells were transfected with LipofectamineTM 2000/3000 (Thermo Fisher Scientific).

The viral supernatant was collected 48 h after co-transfection with the lentiviral expression vector (Ubi-MCS-3FLAG-CBh-gcGFP-IRES-puromycin) and the packaging plasmids pMD2.G and psPAX2 (Addgene, Watertown, MA, USA). Target cells were transduced with the lentivirus in medium containing 8 µg/ml polybrene for 12 h, followed by fresh medium replacement. Stable cell lines were then generated through puromycin selection for further experiments.

### Subcutaneous xenograft model

The subcutaneous tumor xenograft model was established following standard protocols. Briefly, 4 × 10^6^ indicated UM-UC-3 cells were subcutaneously injected into each mouse. Once tumors reached 50–100 mm³, the mice were randomized into treatment groups. Tumor growth was monitored every 3–4 days by measuring orthogonal diameters (a = longest, b = shortest), with volume calculated as V = (a×b^2^)/2. In compliance with ethical guidelines (maximum tumor size: 20 mm diameter, ~ 2000 mm³ volume), the study was terminated at day 28–32, at which point tumors were harvested, weighed, and prepared for histological examination.

### 4D label-free quantitative acetylproteomic and lactylproteomic analysis

The acetylproteomic and lactylproteomic profiling was performed by Beijing Qinglian Biotech Co., Ltd (Being, China). Tissue samples preserved at -80 °C were cryogenically ground in liquid nitrogen with a chilled mortar and pestle. The homogenized material was lysed in buffer (1% Triton X-100, 1× protease inhibitor cocktail, 3 µM trichostatin A, 50 mM nicotinamide) at a 4:1 buffer-to-tissue ratio. After sonication, the lysate was centrifuged (12,000×g, 4 °C, 10 min), and the supernatant was subjected to protein quantification via BCA assay.

#### Protein digestion and processing

Equal protein amounts were adjusted with lysis buffer, then precipitated by stepwise acetone addition (1:1, then 4× volume ice-cold acetone). After incubation at -20 °C for 2 h and centrifugation (4,500×g, 5 min), pellets were washed with ice-cold acetone, air-dried, and dissolved in 200 mM TEAB via sonication. Trypsin digestion (1:50 w/w) proceeded overnight at 37 °C, preceded by reduction (5 mM DTT, 56 °C, 30 min) and alkylation (11 mM IAA, RT, 15 min, dark).

#### Kac- and Kla-modified peptide enrichment

Peptides were dissolved in IP buffer (50 mM Tris-HCl, 100 mM NaCl, 1 mM EDTA, 0.5% NP-40, pH 8.0) and incubated overnight at 4 °C with anti-Kac and anti-Kla antibody beads under gentle rotation. The beads were washed with IP buffer (4×) and deionized H_2_O (2×), followed by elution (0.1% TFA, 3 rounds). Eluates were pooled, concentrated, and desalted using C18 ZipTips (Millipore).

#### LC-MS/MS configuration

Peptides were loaded onto a NanoElute UHPLC system (Bruker Daltonics) in mobile phase A (0.1% formic acid, 2% ACN) and separated over 60 min with a gradient: 7–24% B (0.1% FA in ACN, 40 min), 24–32% B (12 min), 32–80% B (4 min), and 80% B (4 min) at 450 nL/min. Ionization (1.65 kV capillary) and analysis were performed on a timsTOF Pro 2 in PASEF mode (100–1700 m/z MS scan, 2+–5+ precursors, 10 PASEF MS/MS scans/cycle, 24 s exclusion).

#### Data processing

MS/MS spectra were processed in MaxQuant (v1.6.6.0) against the human SwissProt database (20,366 entries) with a decoy database for FDR control. Trypsin/P digestion allowed ≤ 2 missed cleavages (proteome) or ≤ 4 (acetylome/lactylome). Mass tolerances were 20 ppm (precursor) and 0.02 Da (fragments). Fixed (cysteine carbamidomethylation) and variable (lysine acetylation, lysine lactylation, methionine oxidation) modifications were applied. 4D alignment (CCS values) and LFQ (minimum ratio count: 2) were used. For Kac or Kla enrichment, acetylation or lactylation were added as a variable modification, with FDR < 1% for all identifications.

### Co-Immunoprecipitation (Co-IP) and Western Blotting (WB)

Following two washes with ice-cold PBS, cells were lysed in IP buffer (50 mM Tris-HCl, pH 7.5, 150 mM NaCl, 1 mM EDTA, 1% Nonidet P-40, 1% protease inhibitor cocktail) at 4 °C for 30 min. After centrifugation (16,000×g, 4 °C, 20 min), 5% of the resulting supernatant was retained as whole-cell lysate (WCL). The majority of the supernatant underwent immunoprecipitation with target-specific antibodies overnight at 4 °C with constant rotation. 20 µL protein A/G agarose beads (Millipore, IP10) were then introduced, and the mixture was washed five times with chilled IP buffer. The immunoprecipitates were subsequently suspended in 40 µL 2×SDS-loading buffer and heat-denatured at 100 °C for 5 min. Both immunoprecipitated complexes and WCL samples were separated by SDS-PAGE, electro-transferred to PVDF membranes (Millipore, IPVH00010), and blocked with 5% skim milk for 1 h at room temperature before immunoblotting with indicated primary and secondary antibodies.

### Hematein-Eosin (H&E) and Immunohistochemical (IHC) staining

Tissue samples fixed with PFA were sliced into 3 μm sections and mounted on adhesive slides. Following baking at 75 °C for 2 h, the slides underwent dewaxing in xylene (3 × 5 min) and gradual rehydration through an ethanol gradient (100%, 95%, 85%, and 75%; 5 min each). For H&E staining, tissues were sequentially treated with hematoxylin and eosin (1 min each). IHC analysis involved peroxidase inactivation with 3% H_2_O_2_ (15 min), antigen retrieval via microwave treatment in citrate buffer, and blocking with 5% BSA (30 min). Primary antibody incubation was performed overnight at 4 °C, followed by 1-hour room temperature exposure to HRP-conjugated secondary antibodies (OriGene, Beijing, China). Signals were visualized using a DAB detection system (ZsBio), with quantitative analysis conducted using Leica Microsystems LAS v4.12.0 software.

### Immunofluorescence (IF) assay

Cells were seeded onto confocal dishes 24 h before treatment. Following the movement of medium, cell fixation was performed using 4% paraformaldehyde (PFA), with subsequent permeabilization in 0.3% Triton X-100 (15 min). Non-specific binding sites were blocked with 5% BSA prior to overnight incubation with primary antibodies at 4 °C. The samples were then treated with indicated secondary antibodies (1 h, room temperature) and thoroughly washed with PBS (3×). Nuclear counterstaining was achieved using 4′,6-diamidino-2-phenylindole (DAPI, 5 min). Fluorescence images were acquired with a Carl Zeiss LSM880 confocal microscope equipped with a Plan-Apochromat ×63/1.4 NA oil-immersion objective.

## Colony formation assay

A seeding density of 500 cells per well was used in six-well plates, with each group prepared in triplicate. After a 14-day culture period, the resulting colonies were fixed using 4% paraformaldehyde (PFA) for 15 min and subsequently stained with 0.5% crystal violet for 10 min at room temperature. For quantification, colonies with over 50 cells were enumerated under brightfield microscopy.

### Wound-healing assay

Cells were seeded in triplicate in six-well plates and grown to 95% confluency. Using RNase-free pipette tips, uniform scratches were introduced into the monolayer, and detached cells were cleared with PBS washes. Cell migration was assessed by imaging at 0, 12, and 24 h, and the migration ratio was determined by comparing the migrated distance to the initial wound width at 0 h.

### Transwell assay

Cell invasion was assessed using transwell inserts (8 μm, Corning, NY, USA) pre-coated with Matrigel (BD Biosciences). The upper chamber was coated with diluted Matrigel and allowed to polymerize at 37℃ for 4 h. Subsequently, 8.0 × 10^4^ cells suspended in 200 µL serum-free medium were plated in the upper compartment, while the lower chamber was filled with 500 µL complete medium supplemented with 10% FBS. After 10 h of incubation, non-invading cells were removed, and the membranes were fixed in 4% paraformaldehyde (15 min) followed by staining with 1% crystal violet (10 min). The number of cells that migrated to the underside of the membrane was counted under a light microscope.

### Apoptotic assay

Apoptosis was evaluated with an Annexin V-FITC/PI detection kit (40302ES, YEASEN) according to the manufacturers’ instructions. Flow cytometric analysis (NovoCyte, ACEA Biosciences) was performed to determine both apoptotic rates.

### Bulk RNA-seq experiment

Total RNA was extracted using the Total RNA Extraction Reagent (Vazyme, R401-01) according to the manufacturer’s protocol. RNA quality was evaluated using an Agilent 2100 Bioanalyzer with the Agilent High Sensitivity DNA Kit (Personal Biotechnology Co., Ltd., Shanghai). Following Illumina’s standard procedures, cDNA libraries were constructed for high-throughput sequencing. Read alignment was performed with HISAT2 (v2.1.0), chosen for its advanced splice junction detection using reference genome annotations. We employed DESeq2 for differential expression analysis, defining significant differentially expressed genes (DEGs) as those meeting |log2FC| >0.585 (1.5-fold change) with adjusted p-value (FDR) < 0.05. Subsequent functional characterization involved GO term and KEGG pathway analyses was executed via the clusterProfiler R package.

### RNA extraction and real-time quantitative PCR (qPCR) assay

Total RNA was isolated using the Total RNA Extraction Reagent (Vazyme, R401-01) following the manufacturer’s guidelines. cDNA was then synthesized with HiScript III RT SuperMix for qPCR (+ gDNA wiper) (Vazyme, R323-01). Quantitative PCR was performed on a QuantStudio 6 Flex Real-Time PCR System (Applied Biosystems) employing ChamQ Universal SYBR qPCR Master Mix (Vazyme, Q711-02). All primer sequences are detailed in Supplementary Table 1.

### ATAC-seq experiment

A total of 5.0 × 10^4^ cells were initially centrifuged at 500×g for 5 min, washed with 50 µL ice-cold PBS, and spun again under identical conditions. After resuspension in cold lysis buffer (10 mM Tris-Cl pH 7.4, 10 mM NaCl, 3 mM MgCl_2_, 0.1% IGEPAL CA-630), the lysate was processed through sequential centrifugation steps: first at 500 g for 10 min at 4°C to isolate nuclei using a fixed-angle rotor, with careful supernatant aspiration to preserve the pellet. The nuclear fraction was then immediately subjected to tagmentation in a 50 µL reaction mix containing TTBL buffer and TTE Mix V50 (Illumina) at 37 °C for 30 min. Post-tagmentation cleanup was performed with a Zymo DNA Clean & Concentrator-5 kit, followed by library amplification using Q5 Hot Start polymerase with index primers (Vazyme TD202) through 13 PCR cycles (98 °C/10s, 63 °C/30s, 72 °C/1min). Final purification using VAHTS DNA Clean Beads yielded sequencing-ready libraries, which were analyzed by the high-throughput platform (Personal Biotechnology Co., Ltd., Shanghai).

### Isolation of cytoplasmic and nuclear RNA

Cytoplasmic and nuclear RNA were isolated using the Cytoplasmic & Nuclear RNA Purification Kit (Norgen Biotek, NGB-21000) following the manufacturer’s protocol. Briefly, cells were sequentially lysed. The cytoplasmic fraction was collected following lysis with the Cytoplasmic Lysis Buffer. The intact nuclei were pelleted and subsequently lysed with the Nuclear Lysis Buffer. RNA from each fraction was purified through the provided silica-membrane columns, washed, and eluted in nuclease-free water. RNA quality was assessed prior to downstream analysis.

### CUT&Tag analysis

CUT&Tag analysis was performed using the CUT&Tag Kit (Vazyme) following the manufacturer’s instructions. Briefly, 1.0 × 10^5^ cells were captured using ConA magnetic beads. This was followed by sequential incubation with a specific primary antibody, a secondary antibody, and pA-Tn5. After DNA fragmentation and extraction, the sequencing library was constructed via PCR amplification. The library was subsequently sequenced on an HiSeq platform using a paired-end 150 bp (PE150) strategy. The generated sequencing data were analyzed with a cloud-based bioinformatics platform provided by Vazyme.

### Nanopore full-length transcriptome sequencing

Full-length cDNA libraries for Oxford Nanopore sequencing were prepared from 1 µg of high-integrity total RNA (RIN > 8.0) using the SQK-PCS109 kit. Poly(A) + RNA was enriched and subjected to reverse transcription with a strand-switching primer to capture complete transcript sequences, followed by PCR amplification with barcoded primers. After purification and adapter ligation, the library was sequenced on a MinION flow cell (R9.4.1) for approximately 24 h, with real-time base-calling performed via MinKNOW. Differential expression analysis was subsequently carried out using the DESeq2 package, whereby transcripts exhibiting an absolute fold change > 1.5 and an adjusted *p*-value (FDR) < 0.05 were classified as statistically significant.

### RNA Immunoprecipitation assay (RIP)

RIP assay was performed using the Purebinding^®^ RIP Kit (GENESEED, China) following the manufacturer’s instructions. Briefly, 1.0 × 10^7^ cells were subjected to lysis with RIP buffer via freeze-thaw cycles. Meanwhile, primary antibodies or a control IgG (5 µg per sample) were immobilized onto Protein A/G magnetic beads through an overnight incubation at 4 °C. After isolating the immunoprecipitation complexes, the bound RNAs were eluted, extracted, and prepared for subsequent RT-qPCR analysis.

### Statistical analysis

All data were analyzed using GraphPad Prism 10.0 and presented as mean ± Standard Deviation (SD). All experiments were performed with at least three independent biological replicates. Group comparisons were assessed using unpaired two-tailed Student’s t-tests (two groups) or one-way ANOVA with Tukey’s post-hoc test (multiple groups). For all analyses involving multiple comparisons (e.g., post-hoc tests following ANOVA), the *p* values were adjusted using the Bonferroni correction method. *p* value < 0.05 was identified as statistically significant.

## Results

### HDAC2 expression is up-regulated in BCa

**Fig. 1:**
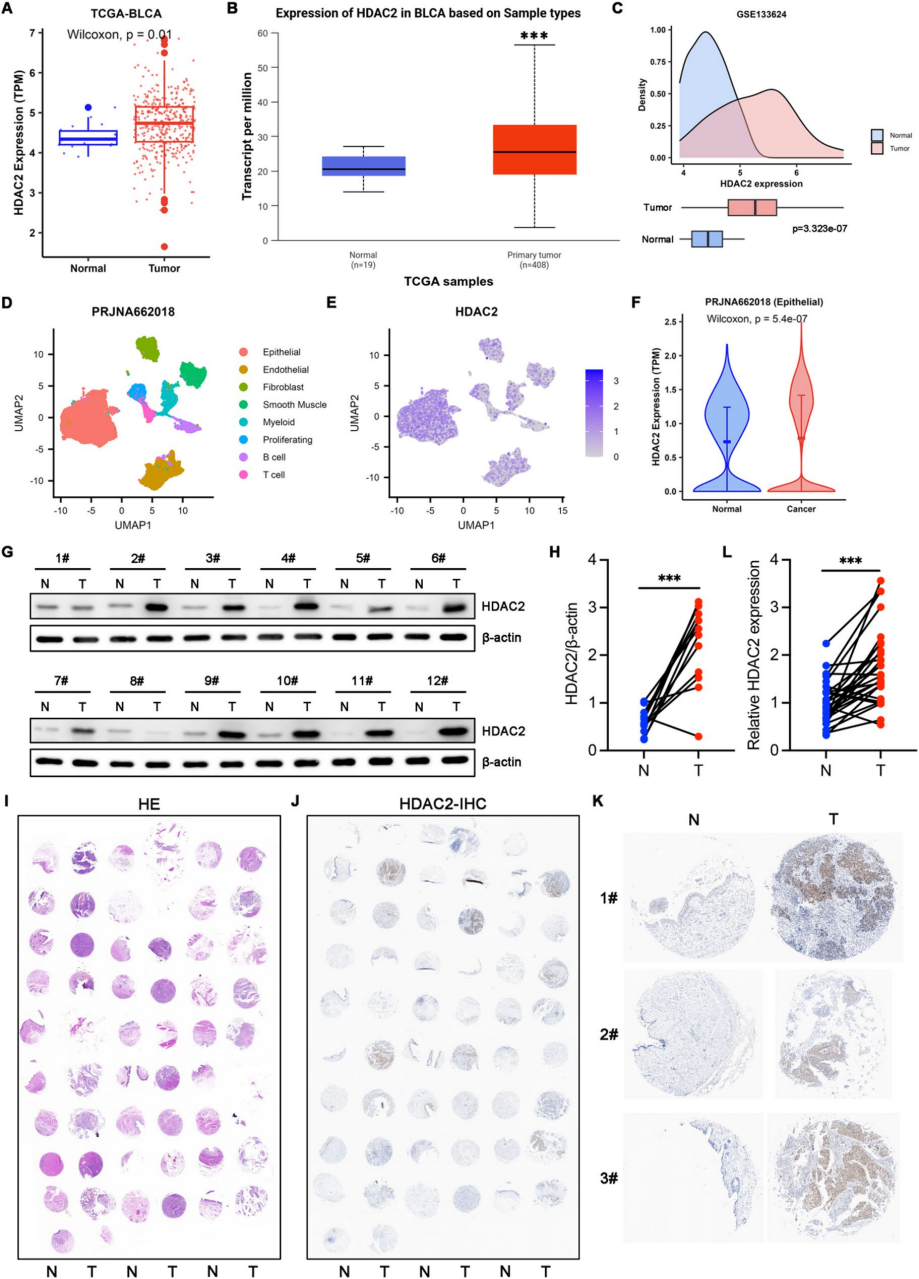
HDAC2 expression is up-regulated in BCa. **A** Expression of HDAC2 mRNA in BCa tissues and paired adjacent normal tissues analyzed using TCGA-BLCA dataset. *p* = 0.01 by Wilcoxon rank-sum test. **B** HDAC2 mRNA expression of BCa and paired adjacent tissues searched through the UALCAN database. ***p < 0.001 by Wilcoxon rank-sum test. **C** HDAC2 mRNA expression of BCa and paired adjacent tissues based on dataset of GSE133624, analyzed by Wilcoxon rank-sum test. **D** Visualization of uniform manifold approximation and projection (UMAP) plots from the scRNA-seq dataset (PRJNA662018). **E** HDAC2 expression dot plots for each cell cluster from the scRNA-seq dataset, as in D. **F** HDAC2 mRNA expression of non-malignant and malignant epithelial tissues in the scRNA-seq dataset (PRJNA662018), which is analyzed by Wilcoxon rank-sum test. **G** and **H** Immunoblotting and quantitative analysis of HDAC2 in twelve paired human BCa tuman specimens (T) and adjacent normal tissues (N). ***p < 0.001 by paired Student’s t test. **I** H&E staining of tissue microarrays from twenty-eight paired human BCa tumor samples and adjacent normal tissues. **J** IHC staining of HDAC2 in tissue microarrays from twenty-eight paired human BCa tuman samples and adjacent normal tissues. **K** Representative images of HDAC2 IHC staining in human BCa tumor samples (T) and adjacent normal tissues (N). **L** Quantitative analysis of HDAC2 IHC staining in twenty-eight paired human BCa tuman specimens (T) and adjacent normal tissues (N). ***p < 0.001 by paired Student’s t test

 To examine the expression patterns of HDAC2 in BCa, we analyzed multiple publicly available databases. Investigations using TCGA database revealed a marked upregulation of HDAC2 in BCa specimens relative to the adjacent non-tumor tissues (Fig. 1A). Furthermore, the aberrant upregulation of HDAC2 mRNA in tumor samples was further confirmed using the UALCAN database (Fig. 1B) and a BCa-related GEO dataset (GSE133624) (Fig. 1C). Analysis of a public single-cell sequencing dataset of BCa (PRJNA662018) revealed that malignant epithelial cells exhibited significantly higher HDAC2 mRNA expression compared to non-malignant counterparts within the epithelial compartment (Fig. 1D-F). To validate these findings, we performed Western blot (WB) analysis on tumor and adjacent normal tissue lysates from 12 paired BCa cases. The results demonstrated a significant upregulation of HDAC2 protein in malignant tissues, with negligible expression in normal tissues (Fig. 1G-H). Moreover, validation via immunohistochemistry (IHC) analysis using tissue microarrays (TMAs) constructed from 28 BCa cases, demonstrated comparable HDAC2 upregulation trends between tumors and matched normal tissues (Fig. 1I-L). These results indicated that HDAC2 expression is significantly upregulated in BCa and may be involved in the malignant behavior of the disease.

### HDAC2 promotes malignant progression of BCa *in vitro*

**Fig. 2:**
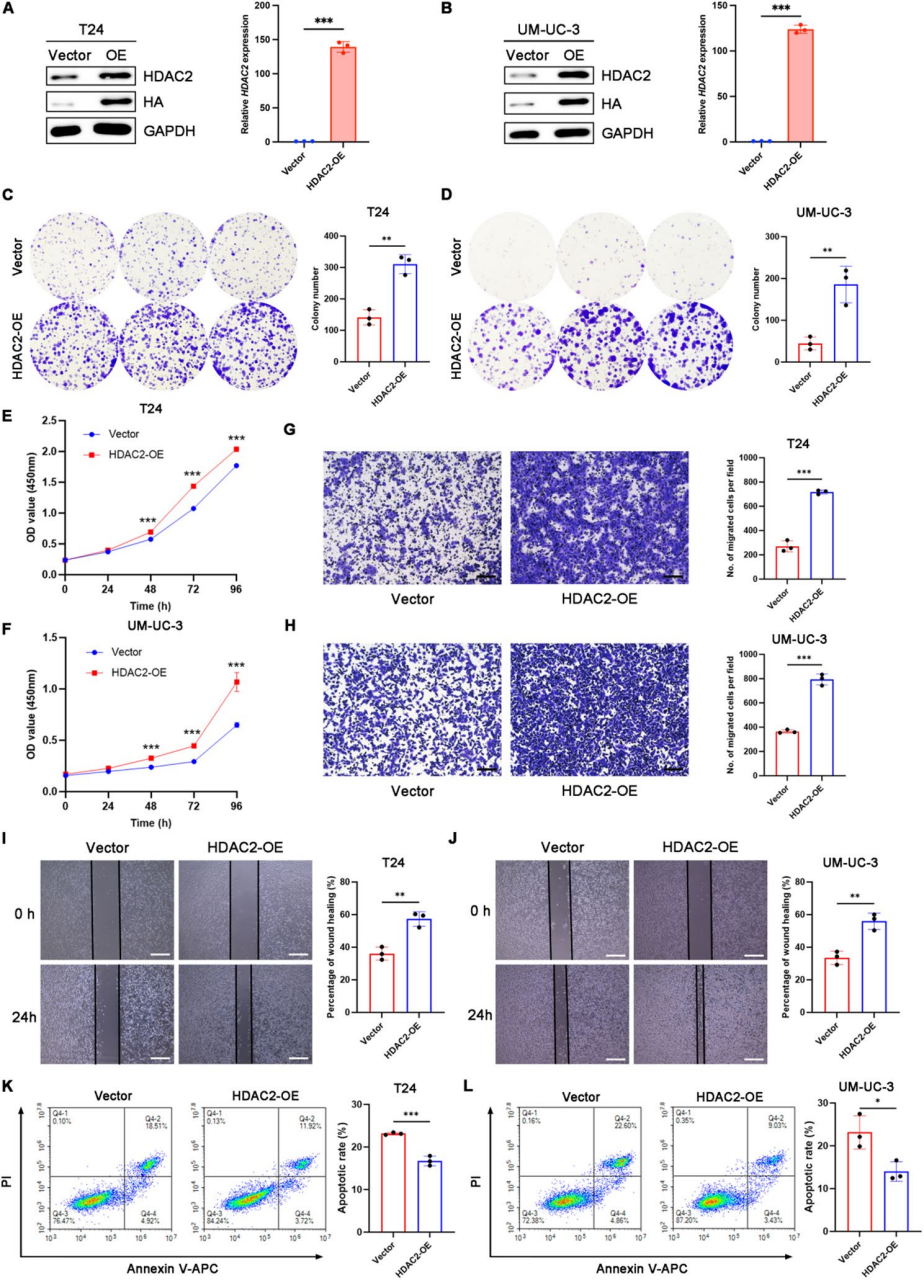
HDAC2 promotes malignant behaviors of BCa cells *in vitro*. **A** and **B** Western blot and qRT-PCR analysis of HDAC2 overexpression in T24 and UM-UC-3 cells. Histogram shows the overexpression efficiency confirmed by qPCR. *n* = 3 biological replicates per group. ***p < 0.001 by Student’s t test. **C** and **D** Colony formation assays were performed in the vector and HDAC2 overexpressing T24 and UM-UC-3 cells. Quantitative analysis of colony numbers was depicted in bar charts. *n* = 3 biological replicates per group. **p < 0.01 by Student’s t test. **E** and **F** The proliferation capacity of vector and HDAC2 overexpressing cells examined by CCK-8 assays. *n* = 3 biological replicates per group. ***p < 0.001 by Student’s t test. **G** and **H** Representative images (Left) and quantified results (Right) of transwell assays in vector and HDAC2 overexpressing BCa cells. Scale bars, 300 μm. *n* = 3 biological replicates per group. ***p < 0.001 by Student’s t test.** I** and **J** Representative images (Left) and quantified results (Right) of wound healing assays in vector and HDAC2 overexpressing BCa cells. Scale bars, 300 μm. *n* = 3 biological replicates per group. **p < 0.01 by Student’s t test. **K** and **L** Flow cytometry was employed to analyze apoptosis in vector and HDAC2-overexpressing BCa cells (left), and the percentage of apoptotic cells was quantified (right). *n* = 3 biological replicates per group. *p < 0.05, ***p < 0.001 by Student’s t test

To elucidate the effects of HDAC2 on BCa cell function, we stably overexpressed HDAC2 via lentiviral transduction in BCa cell lines UM-UC-3 and T24. Both qPCR and WB analyses confirmed significant upregulation of HDAC2 mRNA and protein levels in the overexpression groups (Fig. 2A-B). Compared with cells transfected with vector, HDAC2 overexpression significantly increased the colony-forming capacity of BCa cells (Fig. 2C-D). CCK-8 assays revealed a marked enhancement in cell viability in HDAC2-overexpressing BCa cells (Fig. 2E-F). Additionally, transwell and wound healing assays further demonstrated that HDAC2 overexpression significantly promoted the migratory ability of BCa cells (Fig. 2G-J). The effect of HDAC2 overexpression on BCa cell apoptosis was examined using Annexin V-PI staining kit, which showed significant reductions in both early (Annexin V^+^PI^−^) and late (Annexin V^+^PI^+^) apoptotic cell populations (Fig. 2K-L). These results collectively indicated that HDAC2 overexpression promotes oncogenic functions by enhancing proliferation and migration while suppressing apoptosis in BCa cells.

### HDAC2 contributes to cisplatin resistance of BCa both *in vitro* and *in vivo*

**Fig. 3:**
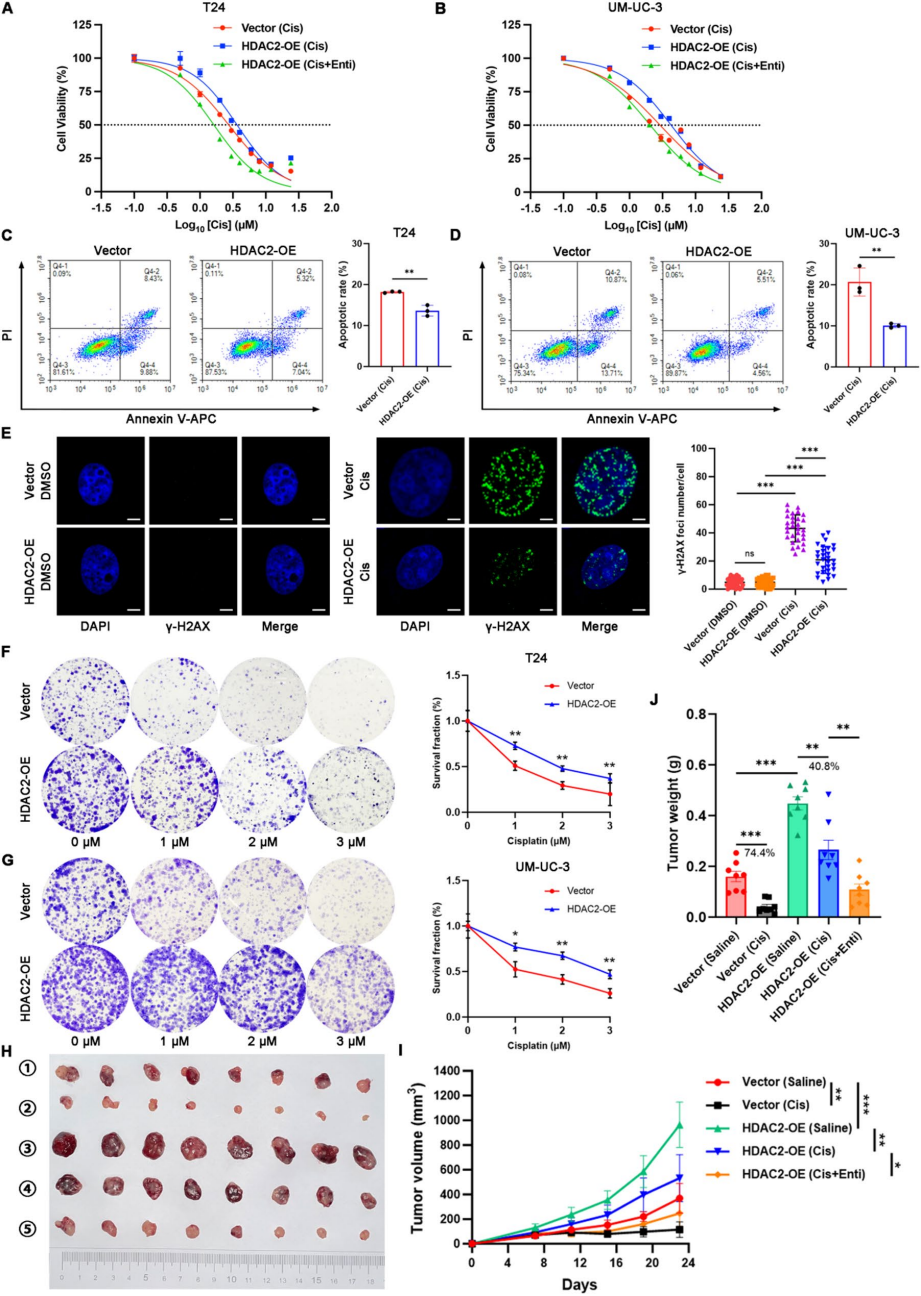
HDAC2 expression is related to chemoresistance of BCa both *in vitro* and *in vivo*. **A** and **B** Cell viability of T24 (**A**) and UM-UC-3 (**B**) cells treated for 48 h in the indicated groups was analyzed by CCK-8 assays, and the IC50 values were calculated. **C** and **D** Apoptotic assays were performed in the indicated cells treated with cisplatin (5 μM) for 24 h. Quantitative analysis of the percentage of apoptotic cells was depicted in bar charts. *n* = 3 biological replicates per group. **p < 0.01 by Student’s t test. **E** Representative immunofluorescence images and quantification data of γ-H2AX foci in the UM-UC-3 cells treated with cisplatin (10 μM) for 24 h. Scale bars, 50 µm. *n* = 30 cells per group. ***p < 0.001 by Student’s t test. **F** and **G** Colony formation assays were performed in the T24 and UM-UC-3 cells treated with indicated concentrations of cisplatin for 7 d. Quantitative analysis of survival fraction was depicted in line charts. *n* = 3 biological replicates per group. *p < 0.05, **p < 0.01 by Student’s t test. **H**-**J** Tumor growth (**H** and **I**) and tumor weight (**J**) of UM-UC-3 subcutaneous model were monitored and measured in the indicated treatment groups. *n* = 8 biological replicates per group. *p < 0.05, **p < 0.01, ***p < 0.001 by Student’s t test

 The above results indicated that HDAC2 overexpression could promote the malignant behavior of BCa cells. Given the established role of HDACs in driving drug resistance across cancers and the status of cisplatin-based chemotherapy as the first-line treatment for advanced bladder cancer (BCa), we investigated the specific involvement of HDAC2 in cisplatin resistance. As the IC50 is a critical indicator of drug sensitivity, we first employed a CCK-8 assay. Results showed that BCa cells overexpressing HDAC2 exhibited reduced sensitivity to cisplatin, whereas treatment with the HDAC inhibitor Entinostat reversed this trend and effectively restored cisplatin sensitivity (Fig. 3A-B). Subsequent apoptotic assays revealed that HDAC2 upregulation decreased the proportion of cisplatin-induced apoptotic cells (Fig. 3C-D). A well-established mechanism of cisplatin is to induce double-strand DNA damage in tumor cells, which can be detected using γ-H2AX as a specific marker. Immunofluorescence staining 24 h after cisplatin treatment revealed minimal DNA damage in both untreated control and HDAC2-overexpressing cells, as expected. However, in the cisplatin-treated group, the number of γ-H2AX foci in HDAC2-overexpressing BCa cells was significantly reduced compared to control cells, suggesting that HDAC2 upregulation could counteract cisplatin-induced double-strand DNA damage (Fig. 3E). In addition, colony formation assays demonstrated that HDAC2-overexpressing cells retained more surviving colonies after cisplatin treatment (Fig. 3F-G). These results demonstrated the impact of HDAC2 on the malignant behavior and cisplatin sensitivity of BCa cells *in vitro*.

To further validate the role of HDAC2* in vivo*, we established a subcutaneous xenograft tumor model using the BCa cell line UM-UC-3 in Balb/c nude mice. We found that HDAC2 overexpression promoted tumor growth *in vivo*, as evidenced by increased tumor volume and weight when compared to the control group. Regarding cisplatin treatment, HDAC2 upregulation also significantly impaired cisplatin-induced tumor growth inhibition, while the combination of the HDAC inhibitor Entinostat with cisplatin reversed the cisplatin resistance induced by HDAC2 overexpression (Fig. 3H-J). Collectively, these findings demonstrate that HDAC2 is a key factor influencing cisplatin resistance in BCa.

### HDAC2 overexpression changes chromatin accessibility and transcriptome in BCa

**Fig. 4:**
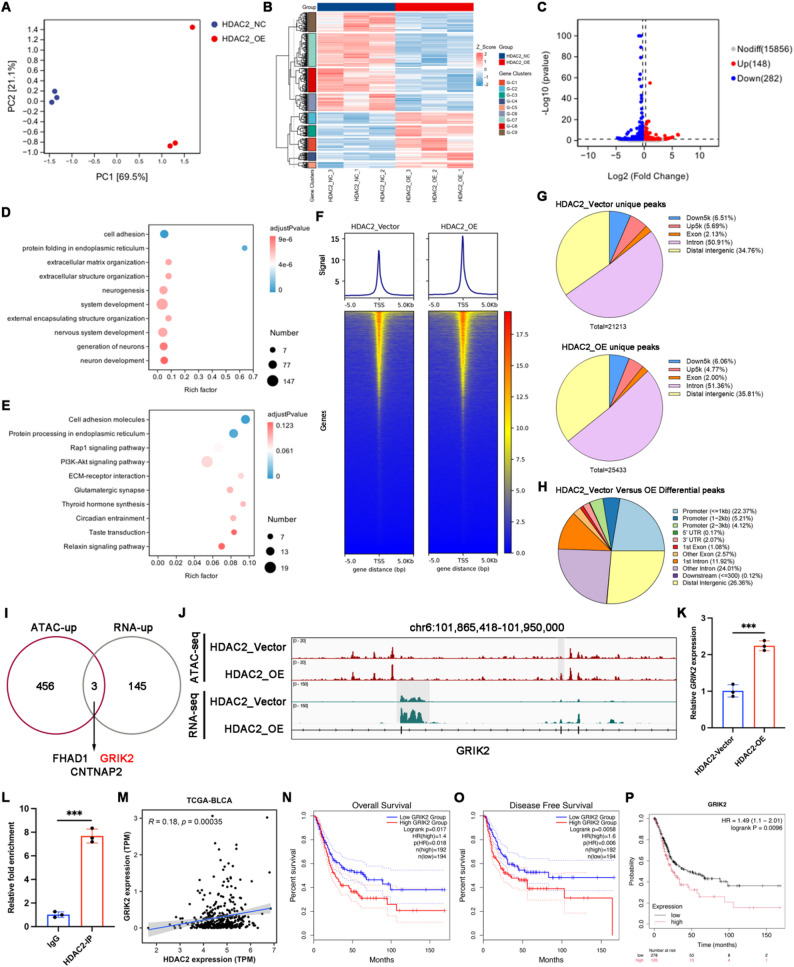
HDAC2 overexpression changes chromatin accessibility and transcriptome in BCa. **A** PCA plot showing distinct transcriptomic profiles induced by vector and HDCA2-overexpressing UM-UC-3 cells. **B** Heatmap displaying the disparities in gene expression between the indicated groups from bulk RNA-seq. **C** Volcano plot of differentially expressed genes between the indicated groups from bulk RNA-seq. **D** GO analysis for differentially expressed genes (DEGs) from bulk RNA-seq. **E** KEGG analysis for differentially expressed genes (DEGs) from bulk RNA-seq. **F** Chromatin accessibility in proximal regions of vector and HDAC2-overexpressing UM-UC-3 cells. **G** Distribution of unique accessible ATAC peaks on genome in HDAC2-vector cells (Upper image) and HDAC2-overexpressing cells (Lower image). **H** Categories of differential open chromatin regions between vector and HDAC2-overexpressing UM-UC-3 cells. **I** Overlapping of the genes proximal to differentially up-regulated accessible regions and genes with up-regulated expression between vector and HDAC2-overexpressing cells. **J** Genomic tracks shows that HDAC2 induces up-regulated accessibility (Up, grey) and expression (Down, grey) at the GRIK2 locus. y axis indicates the RPKM value of each dataset. **K** qRT-PCR measuring the impact of HDAC2 overexpression on GRIK2 gene expression. *n* = 3 biological replicates per group. ***p < 0.001 by Student’s t test. **L** CUT&Tag analysis detecting the binding status of H3K18la in DHRS2 promoter region. *n* = 3 biological replicates per group. ***p < 0.001 by Student’s t test. **M** Correlation analysis of HDAC2 and GRIK2 mRNA expression in the TCGA-BLCA dataset. **N** and **O** The Kaplan-Meier survival curves of overall survival (**N**) and disease-free survival (**O**) for GRIK2 expression in the TCGA-BLCA dataset. **P** Kaplan-Meier plotter database analysis of the relationship between GRIK2 expression and overall survival in BCa patients

To elucidate the downstream pathways regulated by HDAC2 in BCa, we performed RNA-seq on vector and HDAC2-overexpressing UM-UC-3 cell lines to analyze transcriptomic profile alterations (Fig. 4A). A heatmap displayed the distribution of differentially expressed transcripts between the two groups (Fig. 4B). Volcano plot analysis identified 148 upregulated and 282 downregulated genes in HDAC2-overexpressing BCa cells (|FC| >1.5, *p* < 0.05) (Fig. 4C). Gene Ontology (GO) and Kyoto Encyclopedia of Genes and Genomes (KEGG) enrichment analyses indicated that cell adhesion was the most prominently upregulated pathway in the HDAC2-overexpressing group, compared to the vector group (Fig. 4D-E, S1A-D). Moreover, KEGG analysis revealed that HDAC2 overexpression significantly enriched carcinogenesis-related pathways including Rap1, PI3K-Akt, and ECM-receptor interaction, which are critically implicated in BCa tumorigenesis and progression (Figure S1C).

It has been well established that HDAC2 dynamically modulates various histone modifications and maintains a balance with histone acetyltransferases (HATs) to regulate chromatin accessibility and gene expression programs. To further elucidate the epigenetic regulatory mechanisms of HDAC2 in BCa progression, we conducted ATAC-seq analysis on vector and HDAC2-overexpressing BCa cells. The results indicated that chromatin accessibility was globally elevated in the HDAC2-overexpressing group compared to the control, with a pronounced enhancement specifically observed at the transcription start site (TSS) region (Fig. 4F-H). Differential chromatin accessibility peaks specific to the HDAC2-overexpressing group demonstrated significant enrichment for genes implicated in fundamental biological processes and oncogenic signaling cascades (Figure S1E-F). Chromatin accessibility in promoter-proximal regions exerts direct regulatory control over gene transcription. HDAC2 overexpression elicited 782 chromatin binding sites significantly remodeling, with 323 downregulated and 459 upregulated sites. GO and KEGG analyses revealed that the upregulated sites participated in multiple biological processes and signaling pathways, including focal adhesion (Figure S1G-H). By intersecting the upregulated expressed genes from RNA-seq with the upregulated peak-associated genes from ATAC-seq, three overlapping genes were identified: *FHAD1*, *GRIK2*, and *CNTNAP2* (Fig. 4I). Subsequent molecular validation prioritized *GRIK2*, building upon its documented role in regulating stem-like cells and targeting prospects in BCa [27]. Specifically, the *GRIK2* locus demonstrated that elevated transcription in response to HDAC2 overexpression was accompanied by increased chromatin accessibility at this gene (Fig. 4J). qRT-PCR analysis confirmed that HDAC2 overexpression upregulated *GRIK2* mRNA levels (Fig. 4K). CUT&Tag analysis performed with antibodies against HDAC2 also revealed significant enrichment of HDAC2 at the promoter region of *GRIK2* (Fig. 4L). In addition, analysis using the TCGA database revealed a positive correlation between the mRNA expression levels of *HDAC2* and *GRIK2* (Fig. 4M), and higher *GRIK2* expression was correlated with poorer overall survival (OS) and disease-free survival (DFS) in BCa patients (Fig. 4N-O). Kaplan-Meier survival analysis also indicated worse OS in BCa patients with high *GRIK2* expression (Fig. 4P).

Since GRIK2 was identified as a downstream target of HDAC2 regulation, we conducted rescue experiments to determine whether GRIK2 knockdown could counteract the oncogenic effects induced by HDAC2 overexpression. *In vitro* functional assays demonstrated that GRIK2 knockdown significantly suppressed the proliferation, colony formation, and migration capacities of UM-UC-3 and T24 cells. Moreover, GRIK2 downregulation successfully reversed the HDAC2 overexpression-induced enhancement of proliferation (Figure S2A-B), migration (Figure S2C, E, F), and colony formation (Figure S2D, G, H) capacities. Collectively, these findings indicated that HDAC2 overexpression promotes tumorigenic functions by increasing chromatin accessibility, leading to transcriptional upregulation of downstream GRIK2.

### HDAC2 reduces lysine Kac and Kla levels in BCa cells

**Fig. 5:**
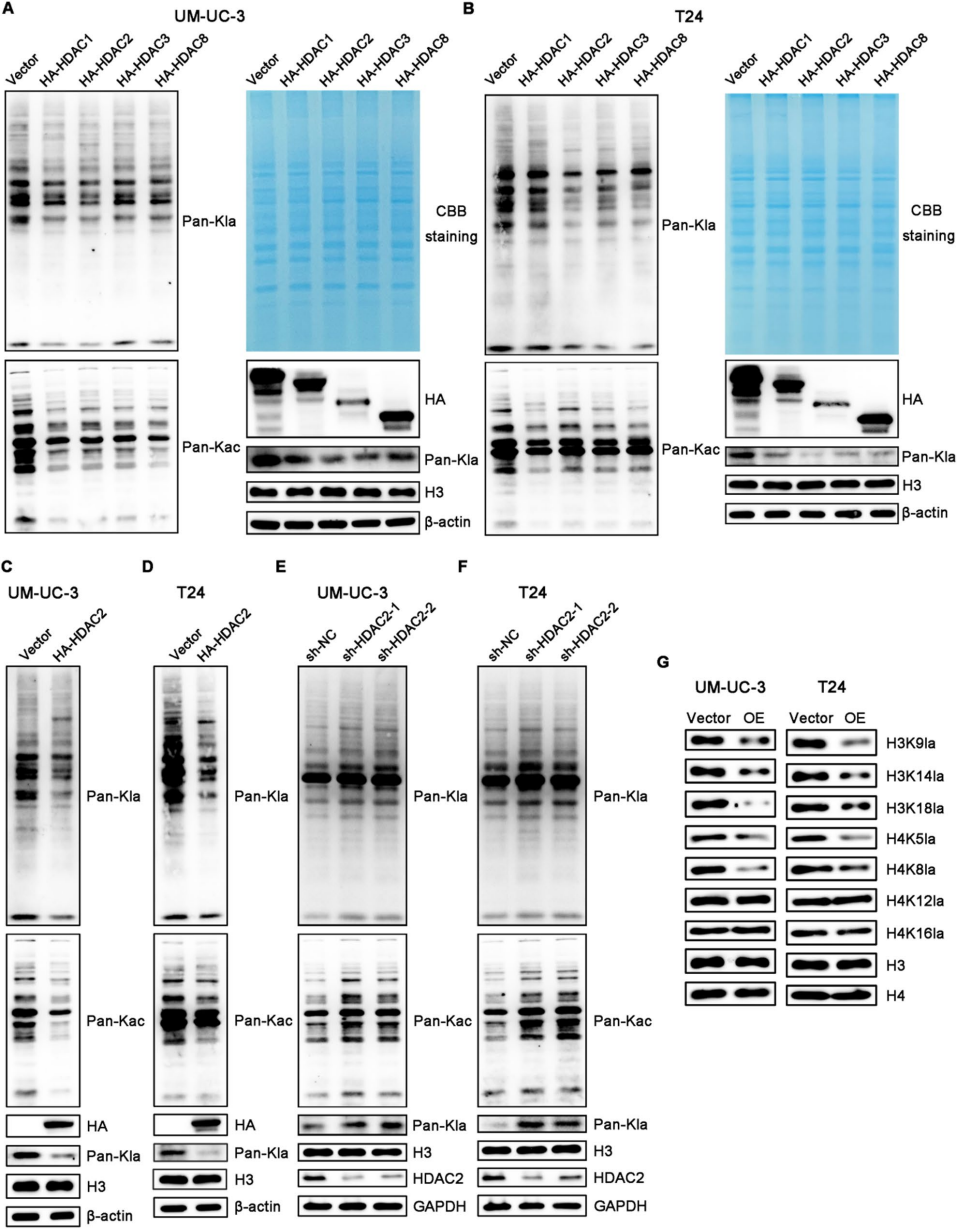
*In vitro* screening of Kla and Kac deacylases among class I HDACs in BCa cell lines. **A** and **B** Western blot analysis of Pan-Kla and Pan-Kac levels after the overexpression of class I HDACs (HDAC1, 2, 3, and 8) for 48 h in UM-UC-3 (**A**) and T24 (**B**) cells. **C** and **D** Western blot analysis of Pan-Kla and Pan-Kac levels in UM-UC-3 (**C**) and T24 (**D**) cells after overexpression of HDAC2 for 48 h. **E** and **F** Western blot analysis of Pan-Kla and Pan-Kac levels in UM-UC-3 (**E**) and T24 (**F**) cells after knocking down of HDAC2 for 48 h. **G** Western blot analysis of Kla levels on histones in UM-UC-3 and T24 cells after overexpression of HDAC2 for 48 h

In addition to transcriptional regulation accessible to chromatin, HDACs is also widely recognized to participate in the processes of PTMs, especially deacetylation and delactylation modifications. However, the functional roles of these enzymes in regulating global Kla and Kac modifications—both on histones and non-histone proteins—remain unclear in BCa. Therefore, we individually overexpressed Class I HDACs (HDAC1, 2, 3, and 8) in UM-UC-3 and T24 cell lines, and assessed the global Kla and Kac modifications by Western blot analysis. As expected, forced expression of each of the four HDACs reduced global Kla and Kac levels. Among them, HDAC2 exhibited the most pronounced effect in decreasing overall Kla levels, while its impact on global Kac was relatively modest (Fig. 5A-B). These results suggest that HDAC2 may serve as a delactylation enzyme in BCa. Moreover, compared to the empty vector, forced expression of HDAC2 exhibited similar results (Fig. 5C-D). In addition, HDAC2 knockdown by shHDAC2 markedly elevated global levels of both Kla and Kac (Fig. 5E-F). Nevertheless, we analyzed the influence of HDAC2 on common histone Kla sites. Consistently, HDAC2 overexpression reduced the modification of H3K9la, H3K14la, H3K18la, H4K5la, and H4K8la, while showing a minor effect on H4K12la and H4K16la. Taken together, these findings suggest that HDAC2 is a critical delactylase in BCa (Fig. 5G).

### Identification of the global lysine lactylome and acetylome in BCa cells

**Fig. 6:**
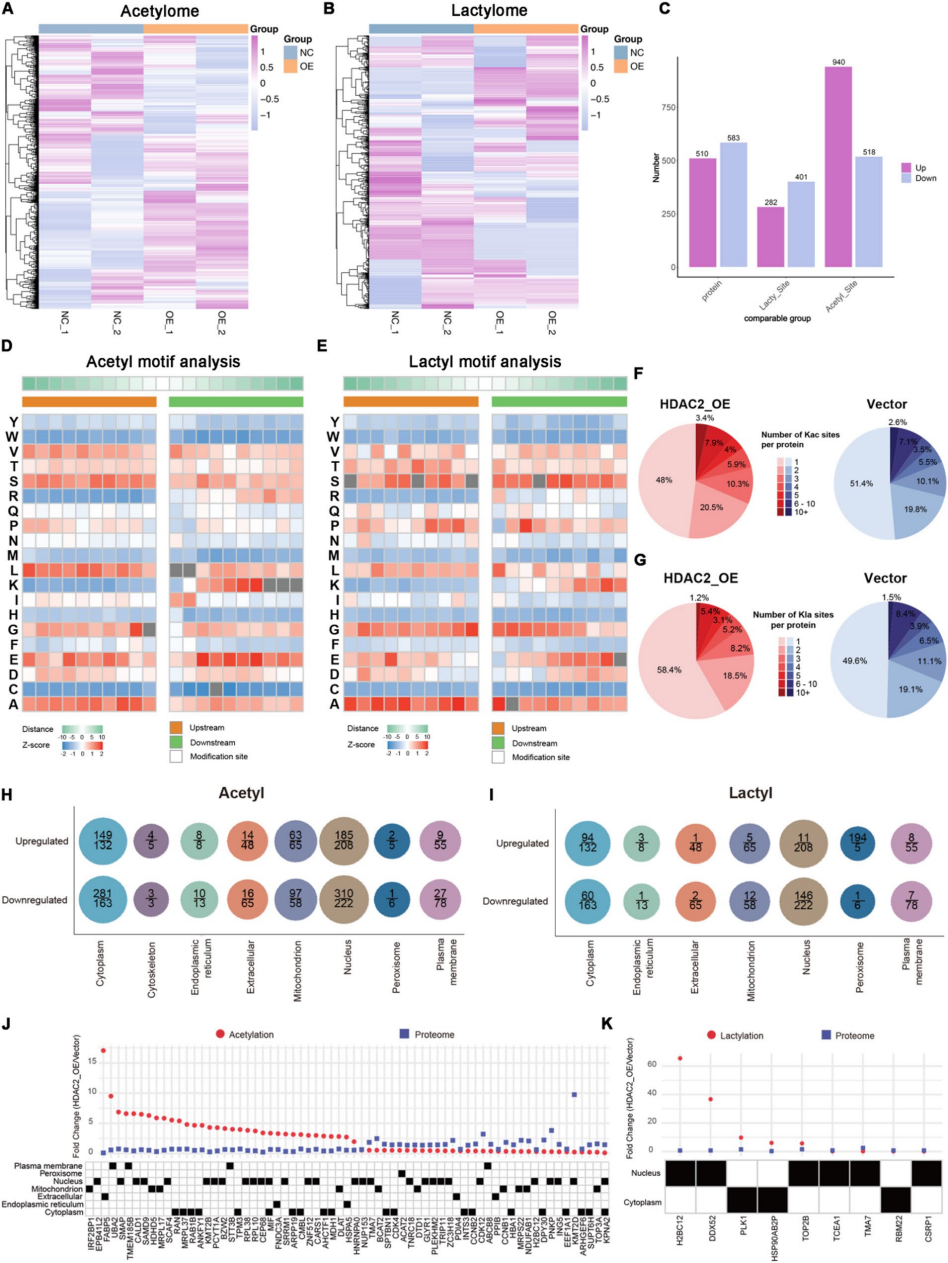
Identification of the global lysine lactylome and acetylome in UM-UC-3 cells by HPLC-MS/MS. **A** and **B** Heatmap displaying the disparities in Kac (**A**) and Kla (**B**) between vector and HDAC2-overexpressing UM-UC-3 cells. **C** Bar charts display the numbers of differentially expressed proteins, lactylated and acetylated sites between vector and HDAC2-overexpressing UM-UC-3 cells. **D** and **E** Heatmap for motif analysis of the different types of amino acids at positions −10 to +10 from the acetylated (**D**) and lactylated (**E**) lysine residue. Different colors represent the preference of each residue (red color denote higher frequency, whereas blue color refers to lower frequency). **F** and **G** Number of Kac (**F**) and Kla (**G**) sites per protein in vector and HDAC2-overexpressing UM-UC-3 cells. **H** and **I** Cellular compartment distribution of differentially Kac (**H**) and Kla (**I**) sites (numbers below the line) and proteins (numbers above the line). **J** and **K** Fold changes for the 67 proteins differed in both the proteome and acetylome (**J**), and 9 proteins differed in both the proteome and lactylome (**K**), as well as their subcellular localization

 To gain insights into the landscape of HDAC2-targeted Kla substrates and its distinct regulatory mechanisms on Kla and Kac patterns in BCa, we performed quantitative analysis of the lactylome and acetylome in empty vector- and HDAC2-overexpressing UM-UC-3 cells using High-Performance Liquid Chromatography-Tandem Mass Spectrometry (HPLC-MS/MS) (Fig. 6A-B, S3A, S3F). A total of 6,588 acetylated and 1,434 lactylated sites were identified, corresponding to 2,302 acetylated and 753 lactylated proteins. Among these, 1,458 acetylated and 683 lactylated sites, as well as 1,129 acetylated and 528 lactylated proteins, were quantified (Figure S3C and S3H), including 940 up-regulated and 518 down-regulated acetylated sites, and 282 up-regulated and 401 down-regulated lactylated sites (Fig. 6C). The results of differential modification site analysis were visualized by volcano plots (Figure S3B and S3G). Kla motif analysis using Motif-X was conducted on all lactylated peptides to investigate the amino acid sequence preference for Kla modification in HDAC2-overexpressing cells. The preference for each residue surrounding the Kla sites was assessed against the human proteome within a 21-amino-acid sequence context using IceLogo heat maps. Alanine and glycine residues, occurring both upstream and downstream of the sites, showed the highest probability of occurrence in lactylated peptides. Tyrosine, tryptophan, methionine, and histidine residues showed the lowest probability (Fig. 6D-E). Cellular compartment analysis demonstrated that the majority of identified and differentially acetylated and lactylated proteins were enriched in the nucleus and cytoplasm, despite their broad distribution (Fig. 6H-I, S3D-E, S3I-J). These findings suggested that HDAC2 might exert critical biological functions by regulating the dynamic modifications of nuclear and cytosolic proteins. In addition, in both vector-transfected and HDAC2-overexpressing UM-UC-3 cells, only 1–3 modification sites were detected for the vast majority of acetylated (78.8%) and lactylated (85.1%) proteins (Fig. 6F-G). Further analysis revealed that HDAC2 simultaneously altered both PTM and protein levels in 67 acetylated and 9 lactylated proteins (Fig. 6J-K), which may serve as critical downstream effectors in the HDAC2-dependent regulatory axis.

### Functional enrichment of HDAC2-regulated differential lysine Kla and Kac

**Fig. 7:**
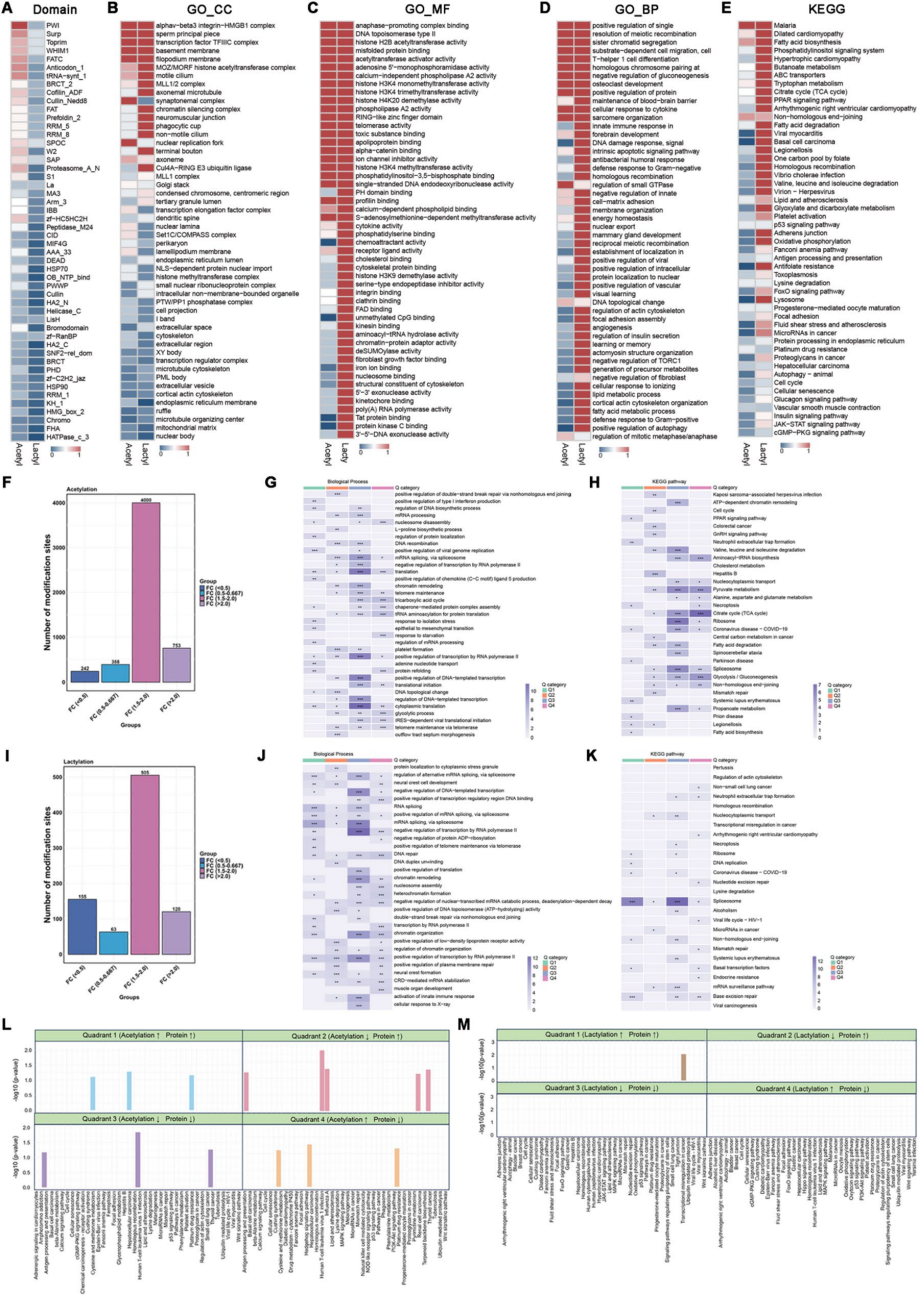
Functional enrichment of lysine lactylome and acetylome in HDAC2-vector and overexpressed UM-UC-3 cells. **A**-**E** Protein domain (**A**), GO_cell component (GO_CC) (**B**), GO_molecular function (GO_MF) (**C**), GO_biological process (GO_BP) (**D**), and KEGG pathway (**E**) analyses of the enrichment of differentially acetylated and lactylated proteins in the four GI cohorts using the DAVID database. Note that 0 and 1 in the legend represents the normalized *p* values, with larger values indicating more significant enrichment. **F** Number of differentially acetylated sites that were classified into four groups according to fold change. **G** and **H** Biological functions and KEGG enrichment analyses of acetylated sites displaying different fold changes (one-way ANOVA, ns: *p* >0.05, *p < 0.05, **p < 0.01, ***p < 0.001). **I** Number of differentially lactylated sites that were classified into four groups according to fold change. **J** and **K** Biological functions and KEGG enrichment analyses of lactylated sites displaying different fold changes (one-way ANOVA, ns: *p* >0.05, *p < 0.05, **p < 0.01, ***p < 0.001). **L** and **M** Barplots showing the highly represented pathways with adjusted *p* value < 0.05 in each quadrant with corresponding alteration of Kac (**L**) and Kla (**M**) sites

Following the characterization of HDAC2’s role in global Kla and Kac, we next investigated the functions of its downstream modified proteins. Protein domain and pathway enrichment analyses revealed significant enrichment of functionally conserved protein domains, cellular components, and molecular functions (Fig. 7A-C). However, the degree of enrichment derived from the same algoithm varied between differentially lactylated and acetylated proteins, suggesting that HDAC2 regulated Kla and Kac in distinct patterns. Moreover, GO and KEGG pathway analyses indicated that HDAC2-regulated differentially modified proteins are involved in metabolism, genomic stability, cellular homeostasis, protein and RNA processing, along with multiple canonical hallmark cancer pathways (Fig. 7D-E). Notably, differentially lactylated and acetylated proteins exhibited specific functional biases, implying that HDAC2 might participate in different biological processes by dynamically modulating Kla and Kac on specific downstream proteins. The aforementioned enrichment analysis of HDAC2-regulated lactylated and acetylated proteins revealed global modification patterns. Building upon these findings, we hypothesized that distinct functional enrichment patterns might exist within specific subgroups of differentially modified proteins. To validate this hypothesis, we stratified each group of modified proteins into four subgroups (Q1–Q4) based on their fold changes. Proteins with modifications of 1.5–2.0 fold changes exhibited a significantly greater magnitude compared to other groups (Fig. 7F and I). GO and KEGG analyses of each subgroup revealed group-specific enrichment patterns. For instance, acetylated proteins in Q3 were predominantly enriched in gene expression regulatory processes–including mRNA processing, splicing, transcription, and translation–while concurrently participating in diverse metabolic pathways (Fig. 7G-H). In contrast, lactylated proteins in Q3 were principally implicated gene expression regulation, as well as cellular homeostasis, chromatin remodeling, and immune responses, exhibiting negligible involvement in metabolic processes (Fig. 5J-K). In addition, we divided the differentially modified proteins, which exhibited significant changes in both the acetylome/lactylome and proteome, into four groups based on ratios of Kla and protein levels. Pathway enrichment analysis revealed distinct patterns among these groups (Fig. 7L-M). Collectively, these findings indicated that HDAC2 differentially regulates Kla and Kac in BCa, potentially exerting distinct effects on tumorigenesis and progression by modulating specific modifications on key proteins.

### HDAC2 differentially regulates lysine Kla and Kac in BCa cells

**Fig. 8:**
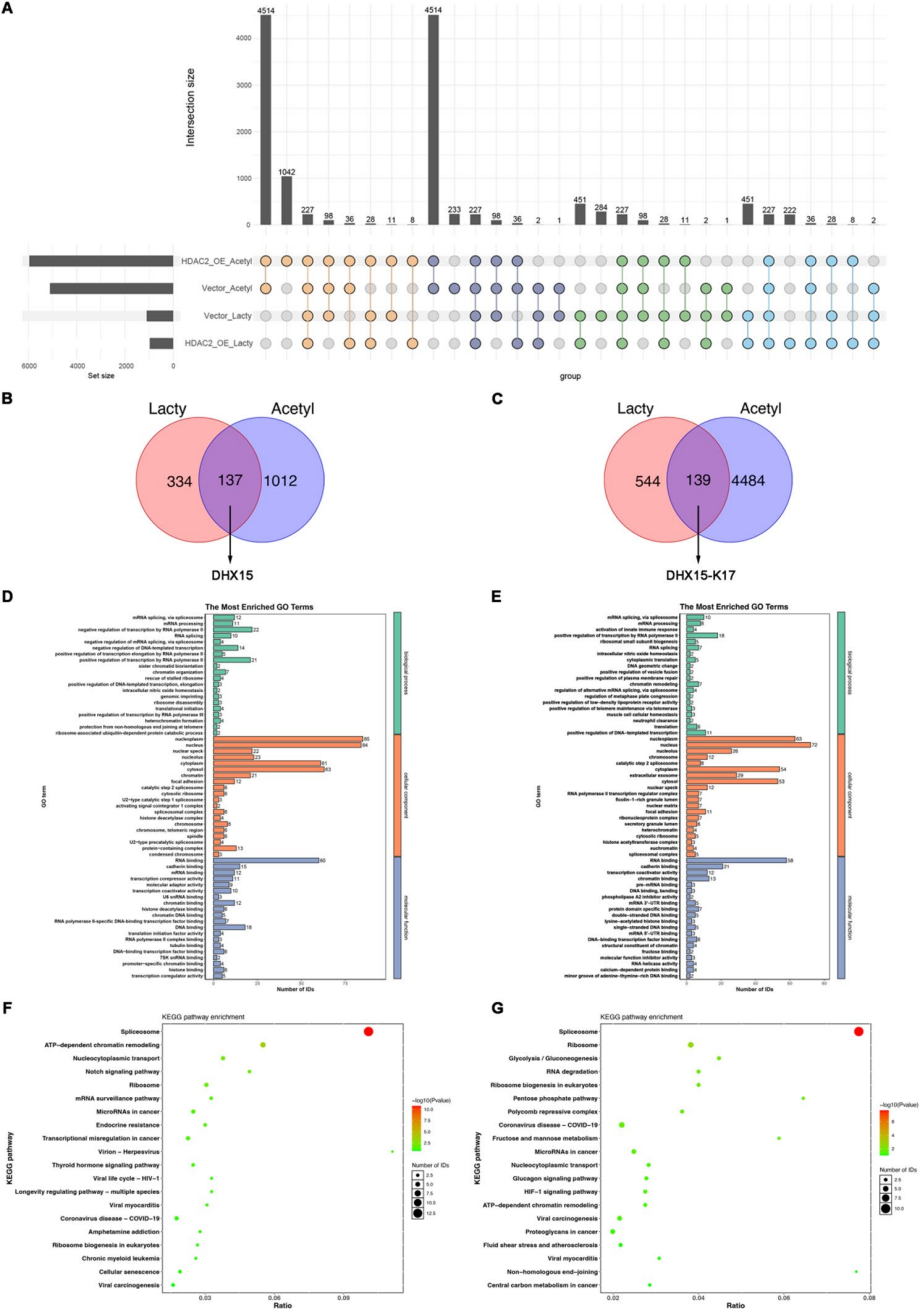
Differential analysis between lysine lactylome and acetylome regulated by HDAC2 in UM-UC-3 cells. **A** Intersection of all Kac and Kla sites identified from vector and HDAC2-overexpressing UM-UC-3 cells. **B** Overlap of proteins exhibiting differential Kla without concurrent Kac changes. **C** Overlap of sites exhibiting differential Kla without concurrent Kac changes. **D** GO functional enrichment analysis of the intersected proteins in B. **E** GO functional enrichment analysis of the intersected sites in C. **F** KEGG functional enrichment analysis of the intersected proteins in B. **G **KEGG functional enrichment analysis of the intersected sites in C

Considering the distinct regulatory patterns of HDAC2 in Kla and Kac in BCa, we subsequently conducted comparative analysis of Kla and Kac sites between empty vector control and HDAC2-overexpressing cells. The overlap rate between these modifications was minimal, suggesting their independent contributions to BCa pathogenesis (Fig. 8A). Given that Kla events in BCa specifically regulated by HDAC2 remained largely uncharacterized, we performed intersectional analysis to define the landscape of proteins and lysine sites that underwent Kla remodeling without accompanying Kac shifts. This approach identified 137 proteins and 139 Kla sites exclusively regulated by HDAC2-driven Kla dynamics (Fig. 8B-C). These HDAC2-targeted Kla proteins and lysine sites showed most significant enrichment in the spliceosome pathway (Fig. 8D-G), suggesting the potential role of HDAC2 in regulating Kla of spliceosome-related proteins in BCa. The initiation and development of BCa are closely associated with aberrant RNA splicing; in particular, mutations or dysreguation of splicing factors can promote BCa through generating abnormal splice variants that activate oncogenic signaling pathways, causing functional loss of tumor suppressor genes, and disrupting critical cellular processes including cell cycle regulation, DNA repair, and epithelial-mesenchymal transition [28, 29]. Notably, DExH-box helicase 15 (DHX15) exhibited the most pronounced Kla downregulation and were thus prioritized for further analysis (Fig. 8B-C).

### HDAC2-mediated DHX15-K17 delactylation facilitates the progression of BCa

**Fig. 9:**
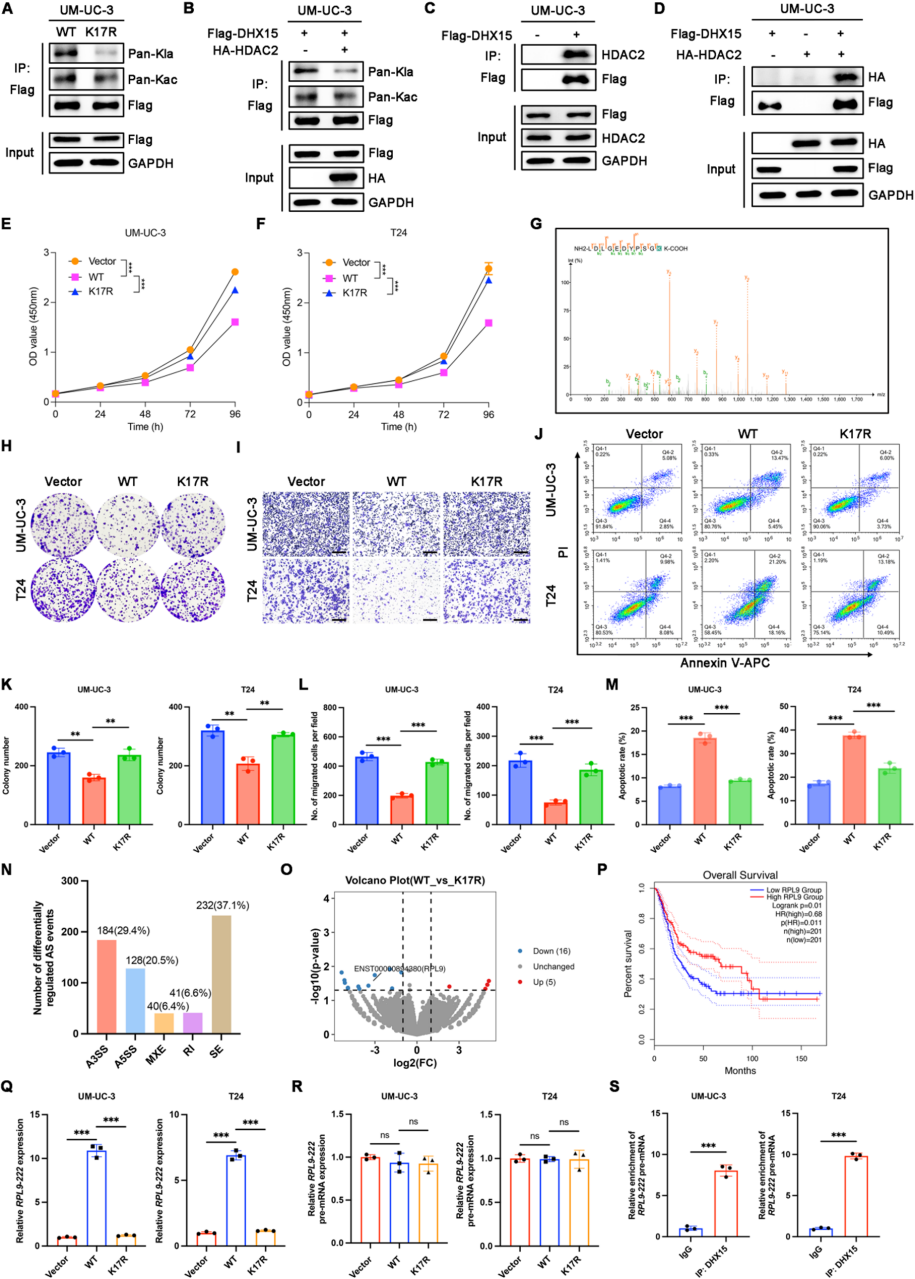
HDAC2-mediated DHX15-K17 delactylation facilitates the progression of BCa. **A** IP assays were performed in UM-UC-3 cells transfected with vector, Flag-tagged DHX15-WT, and K17R for 48 h using anti-Flag antibodies, followed by immunoblotting with Pan-Kla and anti-Flag antibodies. **B** UM-UC-3 cells were transfected with Flag-tagged DHX15, together with HA-tagged HDAC2 for 48 h. IP was performed with anti-Flag antibody and Kla level was detected by WB. **C** Co-IP assay validating the interaction between exogenous Flag-tagged DHX15 and endogenous HDAC2 in UM-UC-3 cells. **D** Co-IP assay validating the interaction between exogenous Flag-tagged DHX15 and exogenous HDAC2 in UM-UC-3 cells. **E** and **F** The proliferation capacity of UM-UC-3 (**E**) and T24 (**F**) cells in the indicated groups examined by CCK-8 assays. *n* = 3 biological replicates per group. ***p < 0.001 by Student’s t test. **G** The MS/MS spectrum and extracted ion chromatograms of DHX15 Kla sites from LC-MS/MS analysis. **H** and **K** Representative images (**H**) and quantified results (**K**) of colony formation assays in the indicated cells. *n* = 3 biological replicates per group. **p < 0.01 by Student’s t test. **I** and **L** Representative images (**I**) and quantified results (**L**) of transwell assays in the indicated cells. Scale bars, 300 μm. *n* = 3 biological replicates per group. ***p < 0.001 by Student’s t test. **J** and **M** Representative images (**J**) and quantified results (**M**) of apoptotic assays in the indicated cells by flow cytometry analysis. *n* = 3 biological replicates per group. ***p < 0.001 by Student’s t test. **N** Differentially regulated AS events between BCa cells tranfected with DHX15-WT and DHX15-K17R plasmids from nanopore full-length transcriptome sequencing data. AS events were classified into five categories: alternative 3’splice site (A3SS), alternative 5’splice site (A5SS), mutually exclusive exon (MXE), retained intron (RI), and skipping exon (SE). **O** Volcano plot illustrating differentially expressed transcripts between DHX15-WT and DHX15-K17R group, with an absolute fold change >1.5 and an adjusted *p*-value (FDR) < 0.05 identified as statistically significant. **P** The Kaplan-Meier survival curve of overall survival for RPL9 expression in the TCGA-BLCA dataset. **Q** qRT-PCR measuring RPL9-222 levels in the indicated cells. *n* = 3 biological replicates per group. ***p < 0.001 by Student’s t test. **R** qRT-PCR measuring nuclear RPL9-222 pre-mRNA levels in the indicated cells. *n* = 3 biological replicates per group. ns: *p* >0.05 by Student’s t test. **S** RIP assays detecting RPL9-222 pre-mRNA enrichment using an anti-DHX15 antibody in BCa cells. RNA enrichment was normalized to a non-targeting IgG control. *n* = 3 biological replicates per group. ***p < 0.001 by Student’s t test

DHX15 was identified as the protein exhibiting the most significant downregulation of Kla levels upon HDAC2 overexpression. As a core component of the spliceosome, DHX15 is critical for pre-mRNA processing [30], yet its role, particularly in the context of cancer and post-translational regulation, remains incompletely understood. Lactylome analysis identified K17 as a Kla site on DHX15, whereas no Kac sites were detected in the acetylome (Fig. 9G). To validate the mass spectrometry data, we mutated lysine residues (K) to arginine (R) to mimic delactylation and overexpressed exogenous Flag-tagged DHX15 wild-type (WT) and mutant (K17R) plasmids in UM-UC-3 cells. Co-immunoprecipitation (Co-IP) and WB analysis revealed that K17R mutations led to a notable decrease of Kla levels on DHX15 while preserving Kac modification levels (Fig. 9A). Furthermore, comparative analysis between the empty vector control and HDAC2 overexpression revealed that HDAC2 significantly downregulated DHX15 Kla while exerting minimal influence on its Kac levels (Fig. 9B).

Classical lactyltransferases and delactylases dynamically regulate Kla by adding or removing lactyl groups through interaction with substrate proteins. We next investigated the interaction between HDAC2 and DHX15. Co-IP analysis demonstrated that exogenous Flag-DHX15 interacted with endogenous HDAC2 in UM-UC-3 cells (Fig. 9C). In addition, co-transfection of Flag-DHX15 and HA-HDAC2 in UM-UC-3 cells confirmed their physical interaction (Fig. 9D).

To preliminarily investigate the role of DHX15 in BCa, we overexpressed empty vector, DHX15-WT or K17R in UM-UC-3 and T24 cells (Figure S4A-D) and found that DHX15-WT overexpression significantly impaired the proliferation capacity of BCa cells. In contrast, overexpression of the K17R mutant abolished this effect (Fig. 9E-F). Furthermore, colony formation (Fig. 9H amd 9 K), transwell (Fig. 9I and L), and apoptotic assays (Fig. 9J and M) demonstrated that reduced Kla of DHX15 enhanced its stemness, migratory capacity, and resistance to apoptosis. These findings indicated that HDAC2-mediated delactylation of DHX15-K17 impaired its tumor-suppressive function, thereby driving BCa progression.

To further elucidate the impact of DHX15-K17la on RNA splicing, we performed nanopore full-length transcriptome sequencing in UM-UC-3 cells overexpressing either DHX15-WT or DHX15-K17R mutant (Figure S4E). Our analysis revealed that exon skipping was the most frequently observed differential alternative splicing (AS) event (Fig. 9N). Differential transcript analysis identified 21 transcripts that were significantly altered in the K17R group compared to the WT group (5 upregulated and 16 downregulated, |FC| >1.5, p < 0.05) (Fig. 9O). Among the eight transcripts that were both differentially spliced and expressed, we focused on a specific transcript of *RPL9* (ENST00000894380, annotated as *RPL9-222* in the ENSEMBL database), which was significantly downregulated and underwent decreased AS events such as A3SS, A5SS and SE in the K17R group. Interrogation of the TCGA-BLCA database indicated that low *RPL9* expression is associated with poor patient prognosis, highlighting its potential clinical relevance (Fig. 9P). qPCR analysis using primers specific for *RPL9-222* confirmed the downregulation of its mature transcript in DHX15-WT cells compared to the vector control, and this effect was reversed by DHX15-K17R mutant (Fig. 9Q). Critically, qPCR analysis of nuclear *RPL9-222* pre-mRNA showed no significant differences across all three groups, nor in any pairwise comparison between them (Fig. 9R). Furthermore, RNA immunoprecipitation (RIP) assays demonstrated that DHX15 directly binds to *RPL9-222* pre-mRNA (Fig. 9S). Finally, functional rescue experiments demonstrated that *RPL9*-222 overexpression reversed the oncogenic phenotypes associated with reduced DHX15 Kla in the context of DHX15-K17R expression (Figure S4F-T).

Collectively, these results demonstrate that HDAC2-mediated delactylation at the K17 site of DHX15 promotes bladder cancer progression, at least in part, by modulating the alternative splicing and downregulating the mature *RPL9* transcript.

## Discussion

In this study, we systematically elucidated the function of HDAC2 in BCa and its underlying mechanisms. HDAC2 promotes malignant behaviors and cisplatin resistance in BCa. As an epigenetic regulator, HDAC2 facilitates BCa progression by enhancing chromatin accessibility to promote downstream *GRIK2* transcription. Furthermore, HDAC2 can function as a delactylation enzyme in BCa, influencing tumorigenesis and progression through regulating the delactylation of spliceosome pathway-related proteins.

Accumulating evidence reveals that HDAC2 facilitates cancer progression in various malignancies, including colorectal cancer [15], hepatocellular carcinoma [31], and breast cancer [32], by coordinating mechanisms such as epigenetic regulation [33], translational activation [34], and immune evasion [35], making it as a critical prognostic marker and therapeutic target. Previous studies have shown that HDAC inhibitors can suppress the activity of class I HDACs, including HDAC2, inducing cell cycle arrest, apoptosis, and DNA damage in BCa cells, thereby impairing tumor progression and overcoming resistance to PARP inhibitors and cisplatin [36, 37]. However, few studies have systematically investigated the role of HDAC2 in BCa. In this study, we analyzed TCGA-BLCA, GEO, and public single-cell transcriptional profiling dataset and found that HDAC2 expression was significantly upregulated in malignant cells compared to non-malignant cells. Additionally, we confirmed that HDAC2 levels were markedly higher in clinical BCa samples than in adjacent normal tissues, suggesting a potential association between HDAC2 and aggressive behavior of BCa. Based on these findings, we conducted *in vitro* functional assays and demonstrated that HDAC2 enhances proliferation, migration, cancer cell stemness, and anti-apoptotic capacity in BCa cells. Given the chemosensitizing effects of HDAC inhibitors in first-line BCa chemotherapy, we further revealed that HDAC2 significantly impaired cisplatin-induced DNA double-strand breaks and enhanced proliferation, stemness, and anti-apoptotic ability following cisplatin treatment, ultimately leading to chemoresistance. Finally, we established a subcutaneous xenograft tumor model in Balb/c nude mice using the UM-UC-3 cell line. The results demonstrated that HDAC2 overexpression promoted tumor growth and compromised cisplatin-induced growth inhibition, and treatment with the HDAC inhibitor Entinostat reversed HDAC2-mediated cisplatin resistance. Collectively, these results highlight the oncogenic and chemotherapy-resistant roles of HDAC2 in BCa.

Considering that the HDAC family dynamically regulates the Kac status of histones and non-histone proteins, influencing chromatin structure and gene expression networks, it is closely associated with epigenetic regulation [10, 38, 39]. Whether HDAC2 can regulate downstream genes through epigenetic mechanisms in BCa remains unknown. Through integrated RNA-seq and ATAC-seq analysis, we found that HDAC2 overexpression increases chromatin accessibility in BCa cells, promoting the transcription of the downstream GRIK2, which was previously reported as a target for bladder cancer stem-like cells (CSCs)/cancer-initiating cells (CICs)-targeting immunotherapy [27]. It is indicated that BCa patients with higher GRIK2 expression exhibit poorer prognosis, and there is a positive correlation between HDAC2 and GRIK2 expression, suggesting that HDAC2 may promote BCa progression by upregulating *GRIK2* transcription. To validate this hypothesis, *in vitro* functional assays confirmed that knockdown of GRIK2 significantly reversed the malignant behavior of BCa cells induced by HDAC2 overexpression. These results demonstrate that HDAC2 promotes BCa progression through an epigenetic mechanism involving increased chromatin accessibility and enhanced transcription of the downstream GRIK2. Our data reveal a unique transcriptional activating role of HDAC2 at the *GRIK2* promoter, which is different from its canonical function as a repressor [33, 40, 41]. We propose several non-mutually exclusive models to reconcile this paradox. Firstly, HDAC2 may inactivate a transcriptional repressor bound to *GRIK2* promoter by deacetylating or transcriptionally downregulating of it, thereby allowing for increased chromatin accessibility. Another model is that HDACs activate *GRIK2* via the recruitment of RNA polymerase II (Pol II) to its promoter, as HDACs are reported to maintain expression of the pluripotent gene network through this mechanism [42]. Furthermore, this activity may not be purely antagonistic to histone Kac but could operate in a dynamic equilibrium with HATs like p300/CBP. Such a cycle of acetylation and deacetylation may be necessary to reset promoters and facilitate productive transcription cycles, underscoring the context-dependent and nuanced role of HDAC2 in gene regulation.

Recent studies have revealed that HDAC2, a crucial enzyme in post-translational protein modification, not only regulates histone deacetylation but also mediates novel PTMs. These include modulating non-histone Kac [43, 44], participating in succinylation [45]of metabolic proteins, and engaging in emerging regulatory networks such as crotonylation [46] and Kla [24, 47]. These discoveries highlight HDAC2’s central role in integrating epigenetic and metabolic regulation. Kla, as a novel post-translational lysine modification, expands the non-canonical metabolic functions of lactate beyond its role as a metabolic byproduct [48–50]. Current research on Kla-modifying enzymes primarily focuses on two key enzyme classes: lactyltransferases (e.g., p300/CBP and AARS1/2) that mediate Kla to promote gene expression and metabolic regulation [17, 51–54], and delactylases (e.g., HDAC1-3 and SIRTs) [24, 25, 55] that remove these modifications, forming a metabolism-epigenetics feedback loop. This intricate regulatory network holds significant research potential. However, the role of HDAC2 in regulating global Kla landscape in BCa remains unclear. Our screening revealed that in BCa, HDAC2 not only mediates deacetylation but also drives the downregulation of global Kla levels. Integrated acetylome and lactylome analyses further demonstrated that HDAC2 overexpression led to 940/282 upregulated and 518/401 downregulated Kac/Kla sites in BCa cells, suggesting HDAC2’s role in suppressing global Kla. To elucidate specific regulatory mechanisms of HDAC2 in Kla, we focused on lactylated rather than acetylated proteins and sites influenced by HDAC2. Functional enrichment analysis highlighted the spliceosome pathway as the most significantly enriched one, implying that HDAC2 may modulate Kla levels of spliceosome-related proteins to impact key processes in BCa. DHX15 emerged as the most prominently regulated spliceosome-associated protein by HDAC2. Further investigations revealed that HDAC2 interacted with DHX15, downregulated Kla at the K17 site and might reverse DHX15’s tumor-suppressive function through regulating alternative splicing of *RPL9*. These findings collectively demonstrate that HDAC2, as a delactylase, dynamically shapes BCa progression by orchestrating the Kla of critical proteins, forming a sophisticated regulatory network.

The identification of HDAC2 as a lysine delactylase positions it as a compelling therapeutic target at the interface of metabolism and epigenetics. Targeting this axis is feasible, as the distinct properties of lactyl-lysine could guide the development of functionally selective inhibitors and enable synergistic strategies in hyper-lactylated environments. However, this approach faces considerable challenges. The primary hurdle is achieving selective inhibition of delactylation without impairing HDAC2’s essential deacetylase function, a lack of selectivity would risk on-target toxicity. Additionally, the pleiotropic consequences of modulating undefined Kla substrates, together with the context-dependent roles of Kla, necessitate precise biomarkers to guide therapy and mitigate unforeseen effects.

## Conclusions

Taken together, we systematically elucidated that HDAC2 facilitates the malignant progression and chemotherapy resistance of BCa via increasing chromatin accessibility to enhancing the transcription of *GRIK2*. Moreover, integrated acetylome and lactylome analyses revealed that HDAC2 regulates the malignant progression of BCa by mediating the delactylation of proteins in the splicesome pathway.

## Supplementary Information

Supplementary Material 1.

## Data Availability

The datasets used and/or analyzed during the current study are available from the corresponding author upon reasonable request.

## Abbreviations

BCa: Bladder Cancer
NMIBC: Non-Muscle-Invasive Bladder Cancer
MIBC: Muscle-Invasive Bladder Cancer
HDACs: Histone Deacetylases
HATs: Histone Acetyltransferases
PTMs: Post-translational Modifications
EMT: Epithelial-mesenchymal Transition
TCGA: The Cancer Genome Atlas
GO: Gene Ontology
KEGG: Kyoto Encyclopedia of Genes and Genomes
OS: Overall Survival
DFS: Disease-free Survival
HPLC-MS/MS: High-Performance Liquid Chromatography-Tandem Mass Spectrometry
H&E: Hematein-eosin
IHC: Immunohistochemical
WB: Western Blotting
Co-IP: Co-immunoprecipitation
IF: Immunofluorescence
TSS: Transcription Start Site
DHX15: DExH-box helicase 15
