# Supplementary material for: HDAC2 as a therapeutic target in bladder cancer: insights into the altered epigenetic regulation and lysine lactylation
Source: J Exp Clin Cancer Res. 2025 Dec 17;45:20. doi: 10.1186/s13046-025-03610-5 (PMC12821908; doi:10.1186/s13046-025-03610-5)
Supplement: Supplementary file 1 — Supplementary Material 1. [file 13046_2025_3610_MOESM1_ESM.docx]

**Supplementary Materials for**

HDAC2 as a therapeutic target in bladder cancer: insights into the altered epigenetic regulation and lysine lactylation

Guanghui Xu^1,2,#^, Shuo Liang^3,#^, Ganlin Hu^4,#^, Wei Zhao^5,6,#^, Yuqin Li^4^, Minghao Zheng^7^, Zhigang Wu^4^, Tianlei Xie^1,2^, Shuting Fang^4^, Shan Peng^8,^ Yongming Deng^1,2^, Yihua Zhou^9*^, Hongqian Guo^1,2*^, Junlong Zhuang^1,2,3,4*^, Wenli Diao^1,2,4*^

This file includes the following subsections:

• Figure S1-4

• Table S1-3


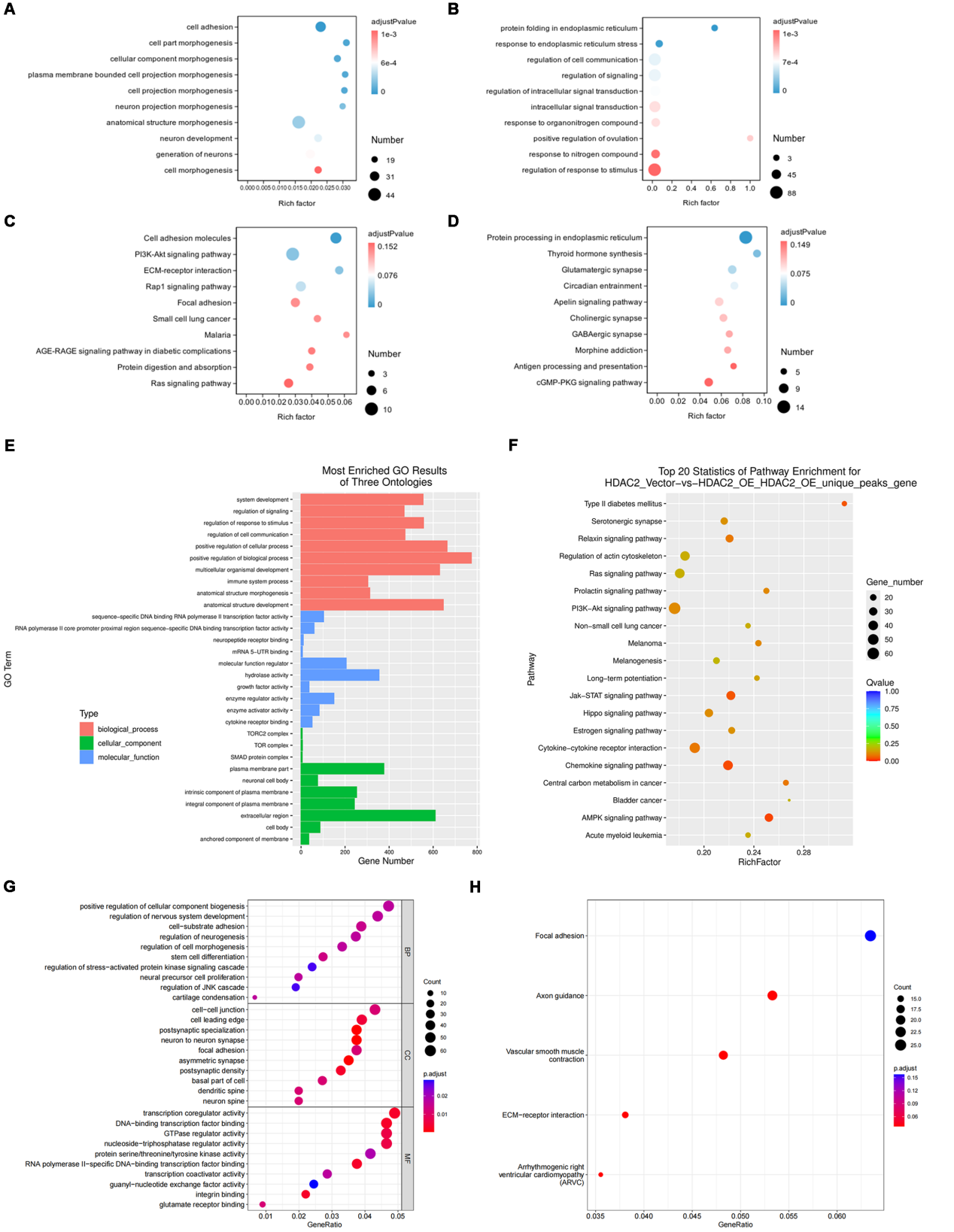


Figure S1. Functional enrichment of HDAC2 expression in RNA-seq and ATAC-seq.

1. GO enrichment analysis for differentially up-regulated genes from bulk RNA-seq.
2. GO enrichment analysis for differentially down-regulated genes from bulk RNA-seq.
3. KEGG enrichment analysis for differentially up-regulated genes from bulk RNA-seq.
4. KEGG enrichment analysis for differentially down-regulated genes from bulk RNA-seq.
5. GO enrichment analysis for unique accessible ATAC peaks of HDAC2-OE group.
6. KEGG enrichment analysis of top 20 pathways for unique accessible ATAC peaks of HDAC2-OE group.
7. GO enrichment analysis for differentially accessible ATAC peaks between HDAC2-vector and HDAC2-OE group.
8. KEGG enrichment analysis differentially accessible ATAC peaks between HDAC2-vector and HDAC2-OE group.


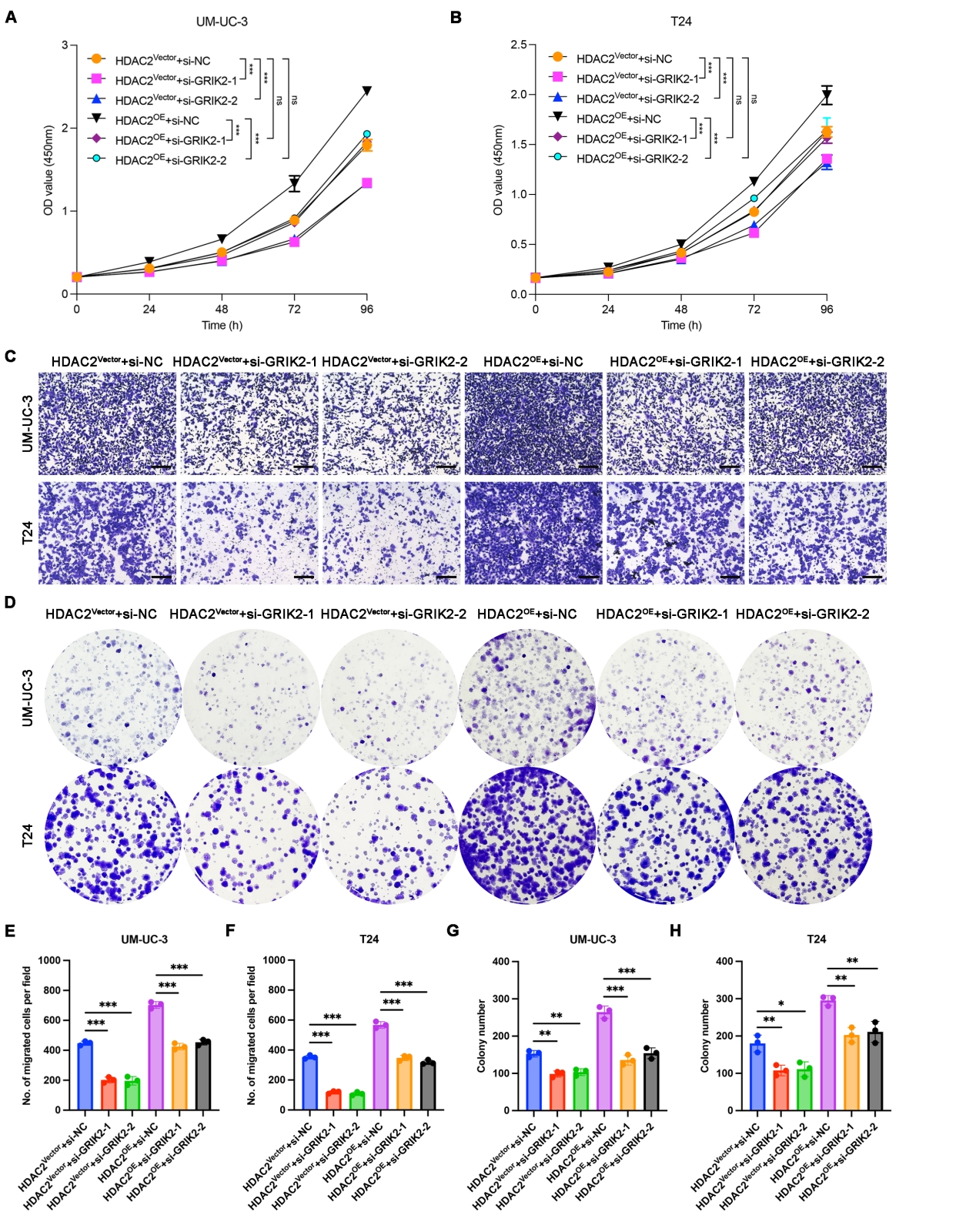


Figure S2. Knockdown of GRIK2 impairs the oncogenic functions of HDAC2.

(A and B) The proliferation capacity of UM-UC-3 (A) and T24 (B) cells in the indicated groups was examined by CCK-8 assays. n = 3 biological replicates per group. ns: *p* > 0.05, ****p* < 0.001 by Student’s t test.

(C, E and F) Representative images (C) and quantified results (E and F) of transwell assays in the indicated cells. Scale bars, 300 μm. n = 3 biological replicates per group. ****p* < 0.001 by Student’s t test.

(D, G and H) Representative images (D) and quantified results (G and H) of colony formation assays in the indicated cells. n = 3 biological replicates per group. **p* < 0.05, ***p* < 0.01, ****p* < 0.001 by Student’s t test.


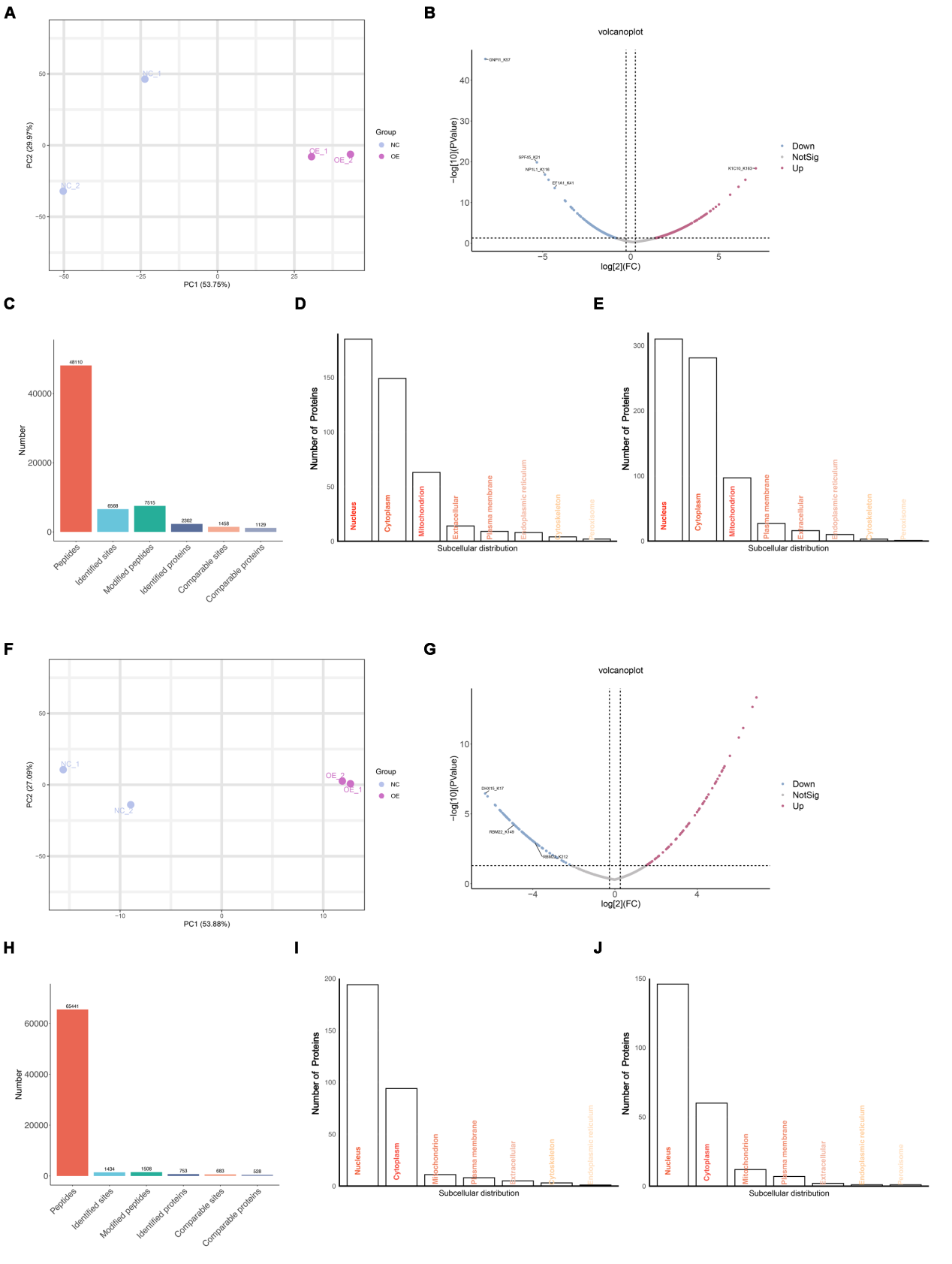


Figure S3. Identification of the global lysine lactylome and acetylome in UM-UC-3 cells by HPLC-MS/MS.

(A and F) PCA analysis of HDAC2-targeted Kac (A) and Kla (F) sites in vector and HDAC2-overexpressing UM-UC-3 cells.

(B and G) Volcano plot of differentially acetylated (B) and lactylated (G) sites between the vector and HDAC2-overexpressing UM-UC-3 cells.

(C and H) Summary of mass spectrometry secondary spectrum in the acetylome (C) and lactylome (H).

(D and I) Subcellular location of up-regulated acetylated (D) and lactylated (I) proteins.

(E and J) Subcellular location of down-regulated acetylated (E) and lactylated (J) proteins.


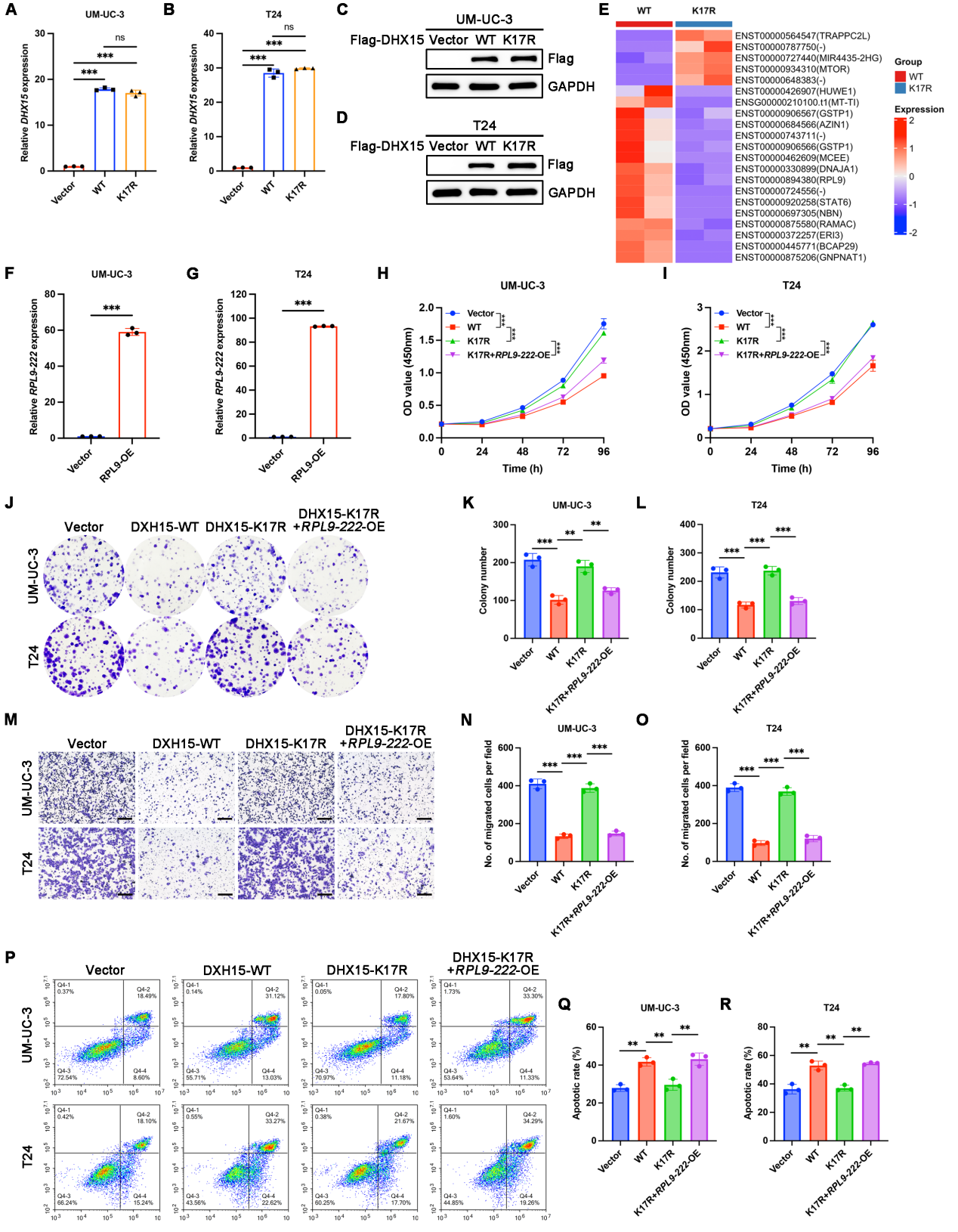


Figure S4. Overexpression of *RPL9*-222 reverses the oncogenic role of DHX15-K17 delactylation.

(A and B) qRT-PCR analysis of DHX15-WT and DHX15-K17R overexpression in UM-UC-3 (A) and T24 (B) cells. n = 3 biological replicates per group. ns: *p* > 0.05, ****p* < 0.001 by Student’s t test.

(C and D) Western blot analysis of DHX15-WT and DHX15-K17R overexpression in UM-UC-3 (C) and T24 (D) cells.

1. Heatmap displaying the differentially regulated transcripts between DHX15-WT and DHX15-K17R group.

(F and G) qRT-PCR analysis of *RPL9*-222 overexpression in UM-UC-3 (F) and T24 (G) cells. n = 3 biological replicates per group. ****p* < 0.001 by Student’s t test.

(H and I) The proliferation capacity of UM-UC-3 (J) and T24 (K) cells in the indicated groups examined by CCK-8 assays. n = 3 biological replicates per group. ****p* < 0.001 by Student’s t test.

(J-L) Representative images (L) and quantified results (M and N) of colony formation assays in the indicated cells. n = 3 biological replicates per group. ****p* < 0.001 by Student’s t test.

(M-O) Representative images (O) and quantified results (P and Q) of transwell assays in the indicated cells. Scale bars, 300 μm. n = 3 biological replicates per group. ****p* < 0.001 by Student’s t test.

(P-R) Representative images (R) and quantified results (S and T) of apoptotic assays in the indicated cells by flow cytometry analysis. n = 3 biological replicates per group. ***p* < 0.01 by Student’s t test.

Table S1 The sequences of qRT-PCR primer

| Gene symbol | Sequence (5'->3') |  |
| --- | --- | --- |
| *ACTB* | Forward primer | CATGTACGTTGCTATCCAGGC |
|  | Reverse primer | CTCCTTAATGTCACGCACGAT |
| *HDAC2* | Forward primer | ATGGCGTACAGTCAAGGAGG |
|  | Reverse primer | TGCGGATTCTATGAGGCTTCA |
| *GRIK2* | Forward primer | TTCAGGCGCACCGTTAAACT |
|  | Reverse primer | GCTCCCATTGGGCCAGATT |
| *GRIK2-L* | Forward primer | GCGGAACTGTAACCTGACAC |
|  | Reverse primer | TCCTCTTCTGGGCAACCATT |
| *GRIK2-S* | Forward primer | CTGCAATGCTCTGGGAGTTC |
|  | Reverse primer | ACGGCTGAGTGAAGAGAAGT |
| *DHX15* | Forward primer | GGGGACCGATGGGAAGGAT |
|  | Reverse primer | TAGCATTTGTTGAAGCTCGCA |
| *RPL9-222* | Forward primer | CTCCGGGTTGACAAATGGTG |
|  | Reverse primer | TCTTGTAACGGAAGCCCAGT |
| *LMNA* | Forward primer | AATGATCGCTTGGCGGTCTAC |
|  | Reverse primer | CACCTCTTCAGACTCGGTGAT |
| *RPL9-222* pre-mRNA | Forward primer | GAGTACGGTGGCGTGATCT |
|  | Reverse primer | AGGTGGGAGAAACACCTGAG |

Table S2 The target sequences of siRNAs

| *HDAC2* siRNA1 | Sense (5'-3') | GCAAAUACUAUGCUGUCAAUUTT |
| --- | --- | --- |
|  | Antisense(5'-3') | AAUUGACAGCAUAGUAUUUGCTT |
| *HDAC2* siRNA2 | Sense (5'-3') | CAGACUGAUAUGGCUGUUAAUTT |
|  | Antisense(5'-3') | AUUAACAGCCAUAUCAGUCUGTT |
| *GRIK2*  siRNA1 | Sense (5'-3') | CUCUAUGGUAAUGAUCGAUUUTT |
|  | Antisense(5'-3') | AAAUCGAUCAUUACCAUAGAGTT |
| *GRIK2*  siRNA2 | Sense (5'-3') | GUUAUCAACAUGCACACAUUUTT |
|  | Antisense(5'-3') | AAAUGUGUGCAUGUUGAUAACTT |

Table S3 Antibody information

| Antibody | Company | Cat# |
| --- | --- | --- |
| Mouse monoclonal anti-GAPDH | Proteintech | 60004-1-Ig |
| Mouse monoclonal anti-β-actin | Proteintech | 66009-1-Ig |
| Rabbit polyclonal anti-DYKDDDDK tag | Proteintech | 20543-1-AP |
| Rabbit polyclonal anti-HA tag | Proteintech | 51064-2-AP |
| Rabbit polyclonal anti-HDAC2 | Proteintech | 12922-3-AP |
| Rabbit monoclonal anti-L-lactyl lysine | PTM Bio | PTM-1401RM |
| Mouse monoclonal anti-Acetyllysine | PTM Bio | PTM-101 |
| Rabbit monoclonal Anti-Histone H3 | PTM Bio | PTM-1001RM |
| Rabbit monoclonal Anti-Histone H4 | PTM Bio | PTM-1015RM |
| Rabbit monoclonal Anti-Lactyl-Histone H3 (Lys9) | PTM Bio | PTM-1419RM |
| Rabbit monoclonal Anti-Lactyl-Histone H3 (Lys14) | PTM Bio | PTM-1414RM |
| Rabbit monoclonal Anti-L-Lactyl-Histone H3 (Lys18) | PTM Bio | PTM-1427RM |
| Rabbit monoclonal Anti-Lactyl-Histone H4 (Lys5) | PTM Bio | PTM-1407RM |
| Rabbit monoclonal Anti-Lactyl-Histone H4 (Lys8) | PTM Bio | PTM-1415RM |
| Rabbit monoclonal Anti-Lactyl-Histone H4 (Lys12) | PTM Bio | PTM-1411RM |
| Rabbit monoclonal Anti-Lactyl-Histone H4 (Lys16) | PTM Bio | PTM-1417RM |
